# Biotechnology of Microorganisms from Coal Environments: From Environmental Remediation to Energy Production

**DOI:** 10.3390/biology11091306

**Published:** 2022-09-02

**Authors:** Nuraly S. Akimbekov, Ilya Digel, Kuanysh T. Tastambek, Adel K. Marat, Moldir A. Turaliyeva, Gulzhan K. Kaiyrmanova

**Affiliations:** 1 Department of Biotechnology, Al-Farabi Kazakh National University, Almaty 050040, Kazakhstan; 2 Institute for Bioengineering, FH Aachen University of Applied Sciences, 52428 Jülich, Germany; 3 Department of Fundamental Medicine, Al-Farabi Kazakh National University, Almaty 050040, Kazakhstan; 4 Department of Applied Biology, M. Kh. Dulaty Taraz Regional University, Taraz 080012, Kazakhstan; 5 Ecology Research Institute, Khoja Akhmet Yassawi International Kazakh-Turkish University, Turkistan 161200, Kazakhstan; 6 Department of Biotechnology, M. Auezov South Kazakhstan University, Shymkent 160012, Kazakhstan

**Keywords:** coal, microorganisms, microbial community, bacteria, fungi, microalgae, biodegradation, bioremediation, humic substances

## Abstract

**Simple Summary:**

Despite the wide perception that coal environments are extreme habitats, they harbor resident microbial communities. Coal-associated habitats, such as coal mine areas/drainages, spoil heaps, and coalbeds, are defined as complex ecosystems with indigenous microbial groups and native microecological networks. Resident microorganisms possess rich functional potentials and profoundly shape a range of biotechnological processes in the coal industry, from production to remediation.

**Abstract:**

It was generally believed that coal sources are not favorable as live-in habitats for microorganisms due to their recalcitrant chemical nature and negligible decomposition. However, accumulating evidence has revealed the presence of diverse microbial groups in coal environments and their significant metabolic role in coal biogeochemical dynamics and ecosystem functioning. The high oxygen content, organic fractions, and lignin-like structures of lower-rank coals may provide effective means for microbial attack, still representing a greatly unexplored frontier in microbiology. Coal degradation/conversion technology by native bacterial and fungal species has great potential in agricultural development, chemical industry production, and environmental rehabilitation. Furthermore, native microalgal species can offer a sustainable energy source and an excellent bioremediation strategy applicable to coal spill/seam waters. Additionally, the measures of the fate of the microbial community would serve as an indicator of restoration progress on post-coal-mining sites. This review puts forward a comprehensive vision of coal biodegradation and bioprocessing by microorganisms native to coal environments for determining their biotechnological potential and possible applications.

## 1. Introduction

Coal is a combustible fossil fuel utilized for different needs, including electricity generation, heating, steel manufacturing, as sources of liquid and gaseous fuels, and precursors in the production of various chemicals/materials [1]. Being a heterogeneous and complex geopolymer, coal and a diverse range of its extracts/derivatives may be subject to microbial attack. Ultimately, the various known microbial metabolic pathways are associated with the wide array of organic substrates, carbon and oxygen content, aromaticity, and relative moisture in coal. Moreover, some natural coals serve as a reservoir of microbial strains able to degrade lignin, having a molecular structure similar to those of coal components [2]. Coals are categorized into several ranks based on depositional, physicochemical, and coalification characteristics that eventually reflect coal microbiology. Coal extraction via surface or underground mining, coal processing/preparation, and energy generation may shape the microbial community structure and functional potential [3].

To date, the available literature body on coal microbiology has generally focused on investigating the physiology and ecology of various microbial community structures/diversity and their activities in coal biodesulfurization processes as well as biogenic coalbed methane production. However, coal environments appear to accommodate diverse microbial catalysis with rich functional potentials to convert coal substrates into value-added products and remediate post-mining sites and industrial deposits.

**Figure 1:**
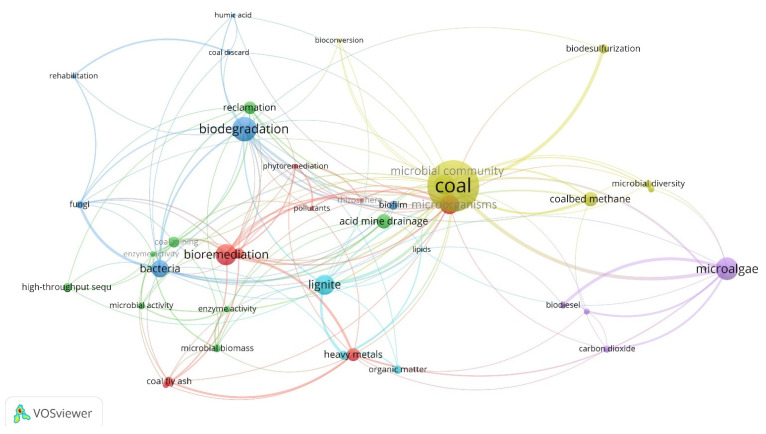
Keyword co-occurrence network visualization map for publications involving “coal* OR low-rank coal* OR lignite*” and “microorganisms* OR microbes*”. Only publications from 1980 to June 2022 were considered. After exporting the publications from the Scopus platform in RIS form, they were analyzed using the keyword co-occurrence function of the VOSviewer software. Here, the top 30 items in terms of the number of occurrences are shown; different circles in the figure represent keywords, and their size indicates the number of times the keywords appear. The lines between the circles indicate that two keywords have appeared together in an article, and the more times they appear, the thicker the line is. On this basis, the main aspects of coal microbiology involve substrates (coal, lignite, low-rank coal, soil, flue gas, and heavy metals), microorganisms (bacteria, microalgae, fungi, and microbial diversity), processes (biodegradation, bioremediation, and biodesulfurization), environments (acid mine drainage, coal mining, wastewater, and sewage sludge), and products (methane, biofuel, and organic matter). By selecting the keyword “microalgae”, as an example, we could observe the connection between this and other keywords, such as coal, flue gas, carbon dioxide, biodiesel, biofuel, etc.

In order to evaluate current research trends and identify the most actively studied topics on coal microbiology, we performed a bibliometric analysis based on keyword co-occurrence. The results were visualized using the VOSviewer software, showing sophisticated interconnections of the topics related to coal microbiology (Figure 1). As one can see from the figure, the major accents currently lay primarily on biotechnological (such as biodegradation, bioremediation, and reclamation) and somewhat less on ecological (microbial community, biofilm, etc.) aspects. One of our goals was to bring these two aspects closer to each other, or rather to emphasize their intrinsic community.

**Figure 2:**
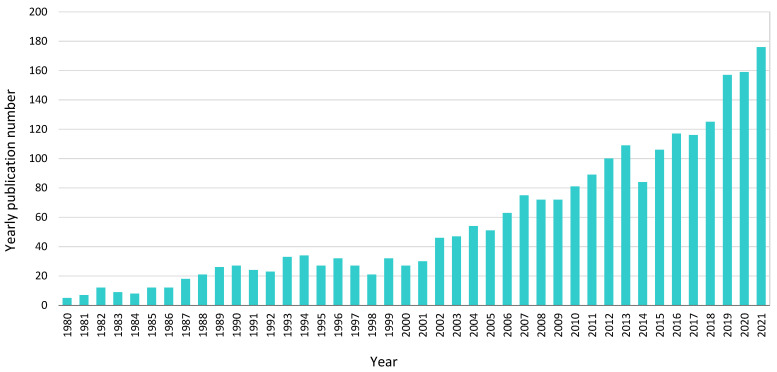
Number of yearly publications about coal microbiology.

The second aspect was the study of the general trend of research by indicating the change in the number of articles involving microbes (microorganisms) in coal (low-rank coal, lignite) over time (Figure 2). Here, the yearly number of publications was counted, which was obtained from the Scopus platform from 1980 to 2021. As can be seen, there has been an upward trend in the number of publications over the years.

Initially, many microbial strains were directly isolated from those environments, which may not be associated solely with coal sources; however, for achieving effective and sustained exploitation and remediation of coal, the relatedness of microbial isolates to the coal sources may be critical. It is intuitively clear that indigenous microbial communities are optimally adapted to their environment in the presence of coal, implying their higher coal degradation efficiency compared with that of exogenous microbial communities. Native strains of microorganisms are already widely documented and are able to grow in culture media with coal as the sole carbon source and solubilize this material, generating humified organic matter [4,5].

Investigation of the microbial communities and main functional genes in coal environments has provided strong evidence that coal sources could be a “seed bank” of various microorganisms with very different functional potentials [6].

In the present comprehensive review based on a systematic literature search, we aimed to take a deep look at the overall functional structure and metabolic potentials of key microorganisms (bacteria, fungi, and microalgae) native to coal environments in the production of value-added compounds and remediation of coal-impacted sites. However, the microbial potentials driving coal biodesulfurization and coalbed methane generation are beyond the scope of this review due to the numerous excellent works published recently [7,8,9].

## 2. Coal Environments as Natural Habitats for Microbial Communities

During surface coal mining, soils/rocks are removed and deposited as “spoil heaps”, which are exposed to runoff, infiltration, acidification, and even spontaneous self-combustion. Spoil heaps vary significantly in their composition and chemistry due to their heterogeneous nature consisting of a mixture of coal seam materials, grained sandstones, clays, and shales. The oxidation of reduced sulfide minerals in coal, brought to the surface during mining processes, leads to acid production, which contributes to acid mine drainage (AMD) [3]. These surface coal-mining-associated environments (spoil heaps and AMD) may provide a suitable habitat for microbial colonization and biological activity due to their complex geomorphology, structural heterogeneity, and variable nutrient contents [10]. AMD-related microbiota inhabit various micro-environments, including water, AMD bed sediments, and macroscopic microbial growths, such as streamers, mats, and slimes [11]. On the other hand, subsurface coal seams and coal beds present an oligotrophic environment for syntrophic assemblages of bacteria and archaea, offering moisture, warmth, and fossilized organic material [12]. Coal-mine-affected soils, along with the spatial distribution of the contamination level and pollution load, can also provide habitats for different microbial communities [13].

The diversity and abundance of microorganisms in coal-affected environments can be a useful bioindicator of post-mining restoration and may complement more traditional agroecological approaches (Table 1). For instance, bacteria belonging to the *Gammaproteobacteria* group may be an accurate bioindicator of potential coal biodegradation [14]. In addition, the presence of bacterial entities, such as *Arthrobacter* spp., *Sinomonas* spp., and *Bacillus* spp., can be a potential biomarker of coal mine spoils [15].

The status of microbial prevalence and community succession in coal environments may be estimated by various methods, such as direct counts, cultivable microbial counts, phospholipid fatty acid analysis (PLFA), total fatty acid profiles (TFAP), and measurements of enzyme activity, CO_2_ uptake, and/or O_2_ consumption [16]. Since the fatty acid composition of microorganisms alters in response to their environment, ratios of specific biomarkers can be employed to assess microbial communities’ physiological and biochemical statuses [17]. Moreover, enzymes have been considered sensitive biomarkers of soil quality in degraded lands and applied to indicate the changes in soil management under different agronomic practices.

Advanced next-generation sequencing methodologies based on metagenomic analyses and 16S or 18S rRNA genes have been used to survey coal mining-associated environments. Through the application of these multiple high-throughput technologies, microbial communities can be characterized in terms of function and structure, and these characteristic profiles can be monitored over time.

## 3. Coal Materials as Substrates for Microorganisms

Coal is a heterogeneous and carbonaceous material with mineral inclusions that microorganisms may be able to attack, degrade, or utilize several different constituents of. In principle, microorganisms can function either in the general breakdown of the coal molecule or in the selective removal of particular components [48,49]. The treatment of low-rank coals with aerobic coal-solubilizing microorganisms produces heterogeneous hydrocarbons and organics of different molecular weights and polarities, with a relatively high oxygen content [50]. Their structural and chemical nature renders low-rank coals more susceptible to microbial modifications, which can be attributed to [51]: (1) higher oxygen content compared with that of higher rank coals, thus providing “biological doorways” for degradation, (2) increased water solubility, which results in improved bioavailability, and (3) structural resemblance to lignin, which allows degradation by lignin-degrading microorganisms.

Several approaches for microbial coal modification have been proposed [52]. Microorganisms may attack coal by focusing on carbonaceous matter and/or interspersed inorganic materials. One approach is depolymerizing the coal polymer, breaking various key links—this could provide the basis for liquefaction. A second approach would be reducing the oxygen content through reducing C=O to CH_2_ or decarboxylation to CO_2_—this could improve the calorific value. A third would be the removal of sulfur, nitrogen, or metals from the coal before firing, which would reduce unwanted emissions.

The pilot observations reported in the 1980s initiated intensive research, leading to a deeper understanding of the parameters and strategies of coal degradation [4]. To date, many studies have stated three principal mechanisms of coal biodegradation/bioconversion: solubilization, depolymerization, and utilization [53,54,55]. However, a number of other terms are often used interchangeably to describe different stages in coal biodegradation/bioconversion, such as solubilization, liquefaction, depolymerization, utilization, decolorization, etc.

Coal ***solubilization*** represents a nonenzymatic dissolution and occurs at an alkaline pH in the presence of alkaline substances, chelators, and surfactants, yielding black liquid. Coal ***depolymerization*** is mediated by enzymes that function at pH levels below 6. Lignin-degrading oxidoreductases and certain hydrolases cleave linkages that maintain the 3D structure of coal and release substances with lower molecular masses [56]. Coal ***utilization*** consists of its biodegradation by various bacteria and fungi, which use components of the mobile part of lignite as carbon sources.

In general, microbial coal transformation is summarized and denoted as the ABCDE system (A = alkali, oxidative; B = biocatalysts; C = chelators; D = detergents; and E = esterases) [2,53]. In most bacteria and actinomycetes, alkaline action and chelation play the primary role, while enzymes play the most crucial role in fungi. Several microorganisms with distinctive physiological characteristics were found to exploit either one or a combination of these mechanisms.

A. alkaline substances (ammonia, biogenic amines, peptides, and their derivatives) and chelators are involved in the microbial solubilization (liquefaction) of coal. These non-enzymatic substances are produced by fungi and bacteria using the organic acids of the medium and increase oxidation by neutralizing the carboxylic acids present in coal, ultimately resulting in coal solubilization.
B. coal depolymerization and solubilization can be achieved through catalytic metabolism, especially with lignin-degrading enzymes, because the structure of low-rank coal is very similar to that of lignin. These enzymes play a critical role in humic acid depolymerization by breaking the covalent bonds within the coal macromolecule. These enzymes can be divided into oxidative (lignin peroxidase, manganese peroxidase, and laccase) and non-oxidative (esterases). Numerous microorganisms (*Penicillium* sp., *Trichoderma* sp., *Bacillus* sp., *Mycobacterium* sp., *Acinetobacter* sp., *Enterobacter* sp., *Rhodococcus* sp.) have been documented to secrete ligninolytic enzymes on culture medium containing coal. Saprotrophic fungi and, in particular, ligninolytic microorganisms may act as biocatalysts for coal transformation [4].
C. chelating agents (e.g., oxalic acid, salicylic acid, and triethylamine) secreted by fungi can react with the metal ions (calcium, iron, and magnesium) in coal and depolymerize its molecular structure, resulting in the generation of small water-soluble molecules.
D. detergents (surfactants) enhance coal solubilization/dissolution by promoting the absorption of biological enzymes on the coal’s surface and by reducing surface tension. In addition, surfactants can also shift the reaction sites of certain enzymes, which may lead to higher coal biodegradation rates.
E. like oxidases, non-oxidative esterases also play a large role in coal degradation. These enzymes are mainly produced by Gram-negative and Gram-positive soil bacteria and can hydrolyze coal polymers by the cleavage of ester or ether bonds.

Biological degradation/conversion of coal sources offers an alternative clean strategy for exploiting massive coal discards/dumps. Several non-fuel options for low-rank coal utilization have been prospected, such as the extraction of soil amendments/conditioning agents, organic moieties, and chemical feedstock for the subsequent generation of alternative fuels.

### 3.1. Peculiarities of Coal Degradation by Bacteria

A variety of bacterial species have been studied for their coal biosolubilization abilities, as they dissolve coal as an energy source for growth. Short-term cultivation, a faster conversion rate, and easier operation enable bacteria to achieve maximal coal degradation under standard temperature and pressure conditions [5,53,57]. Many recent studies suggest that indigenous bacterial isolates from coal environments (coal residues, coal-mining soils, and coal tailing water) have greater coal-solubilizing ability than exogenous microbial communities (Table 2).

The biodegradation of coal may occur faster when the substrate is oxidized; therefore, many studies often employ either brown coal (lignite) or leonardite with different proximate/ultimate compositions. As mentioned above, depending on the type of mechanism involved in the studies, coal biodegradation can be described in many ways, such as biotransformation, biosolubilization, bioconversion, demineralization, decomposition, and bioliquefaction. The rates and extent of coal biodegradation depend upon several factors, including the rank and origin of the coal, type of microbial strains, mode of coal treatment/processing, etc.

**Figure 3:**
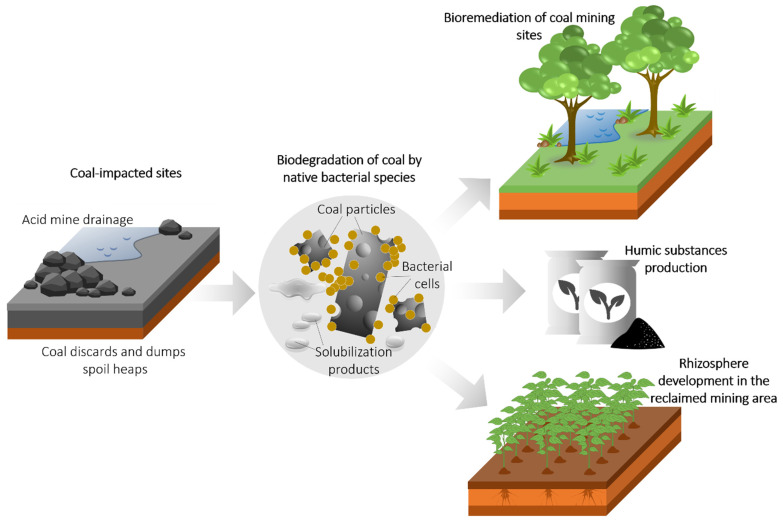
Exploiting indigenous “microbial cocktails” to degrade/convert coal from coal-impacted sites may have multi-faceted advantages in agricultural productivity and environmental sustainability, i.e., the bioremediation of post-coal mining sites, production of humified organic substances, and development of plant growth-stimulating bacteria. More detailed information is provided in the following sections.

Coal biodegradation by microbial isolates is being intensively studied to develop effective rehabilitation and revegetation strategies for coal mining areas and discard dump sites (Figure 3). Such isolates, so-called “microbial cocktails” [51] native to coal environments, can presumably be the best tools for obtaining released humified organic matter from coal for subsequent agricultural applications.

### 3.2. Coal Degradation by Fungi

Primarily, coal degradation by fungal species differs from that by bacteria in terms of the nature of the released organics. Indeed, while the bacterial degradation of coal results in the generation of a mixed pool of organics, including aromatics and aliphatics, the fungal degradation machinery involves mainly polyaromatic hydrocarbons, single-ring aromatics, aromatic nitrogen compounds, and a minor fraction of aliphatics [74]. The elucidated operative mechanisms behind coal solubilization and depolymerization by fungi include both enzymatic (hydrolases; peroxidases, viz., manganese peroxidase and lignin peroxidase; and phenol oxidases, viz., laccases) and non-enzymatic (alkaline metabolites, surfactants, and chelators) agents [75].

A variety of coal-native fungal species have been reported, which efficiently modify the coal matrix and liberate fractions with different molecular weights (Table 3). The indigenous fungal species are preferable to exogenous species because they are more likely to fit into complex coal environments and are well-adapted climatically. In most studies, the fungal strains playing a crucial role in effective coal degradation were isolated from decaying wood around coal mines, coal–soil mixtures, coal mining sites, and purely coal environments.

One of the prospects that can be derived from the data summarized in Table 3 is the use of fungal strains in coal degradation for agricultural sustainability and environmental safety. The application of coal treated with fungal strains has been extensively reported by Gokcay et al. and in other studies devoted to chemical feedstock processing and the production of humic substances for soil conditioning [76].

## 4. Bioremediation of Contaminated Sites by Native Microorganisms

Coal remains the largest fuel source for power generation worldwide, comprising around 40% of global energy production [96]. Mining activities produce a high volume of mine discards and tailings, causing soil erosion, heavy metal contamination, and acid mine drainage. Furthermore, land degradation due to coal mining alters biogeochemical and hydrological cycles, posing severe environmental and health risks in vast mining operations areas [97].

Land devastation in coal mining sites is well documented and emphasizes the need to invest focused effort in developing sound rehabilitation technologies [98,99]. Various traditional chemical approaches have been proposed to restore contaminated mining soils. However, their effectiveness and cost-efficiency are still debatable [100,101,102]. Bioremediation and bioreclamation are increasingly being considered as the primary choice for contaminated site recovery worldwide due to economic, environmental, and safety reasons [4,103].

While the exact biochemical and molecular mechanisms involved in coal biodegradation under ambient conditions remain to be better elucidated, there certainly appears to be a contribution of microbial solubilization [104], oxidation [105], and liquefaction [5,93] that demonstrate the potential to use microorganisms in effective rehabilitation strategies.

### 4.1. Bioremediation of Coal Mining Areas by Microorganisms

Several microbial cultures have been isolated from coal sources (slurries and disposal of coal tailings), screened for coal biodegradation competence, and characterized. Hamidović et al. [97] isolated and identified autochthonous lignite mine spoil bacteria and evaluated their potential in the bioremediation of mine-overburdened soil. The findings of that study also illustrated the soil fertility potential of recovered native species *Bacillus simplex* and *Bacillus cereus*. In a study by David et al. [5], 45 bacterial strains were isolated from the coal sludge, and four strains belonging to *Cupriavidus* sp., *Pseudomonas* sp., and *Alcaligenes* sp., were further evaluated for their coal-degrading activity. The observed ability of these strains in coal depolymerization may be attributed to the aggressive degradation of aromatic compounds (phenol, toluene, benzaldehyde, benzoic acid, and indole), since these compounds have been widely used as models for coal degradation.

The colonization and oxidative metabolism of discarded coal by fungal strains (*Fusarium oxysporum*, *Paecilomyces farinosus*, *Lentinula edodes Trametes versicolor*, and *Phanerochaete chrysosporium*) isolated from coal environments are also well-documented [106,107]. Another fungal organism, *Neosartorya fischeri*, could rapidly colonize and use various complex organics, highlighting its biotechnological potential for application in rehabilitating recalcitrant substrates [95,108].

Apart from using individual microbial isolates, consortia-based remediation could be an attractive alternative for enhancing the rehabilitation of mining sites. Like crude oil and petroleum, coal is a complex hydrocarbon material that usually requires cooperation between species or assemblages of microbial populations to be degraded [109,110]. Specific microbial taxa within a complex consortium demonstrate a different level of substrate specificity proliferating on particular coal fractions. Detman et al. reported that the formation of microbial consortia and their synergistic interactions dramatically enhance lignite degradation [64]. According to Maka et al., mixed cultures of *Bacillus* strains sufficiently degraded crude lignite within two weeks [111]. In a study by Olawale et al. [57], the most successful coal-degrading consortia contained either *Serratia* sp. ECCN 24b or *Exiguobacterium* sp. ECCN 21b, or both, and reduced the coal substrates’ masses by ~10% and ~30%, respectively. Indeed, in another study by Mohanty et al., species of the *Exiguobacterium* were able to actively utilize n-alkanes (C9–C26) [112], while *Serratia* spp. were identified by Benedek et al. as hydrocarbon degraders [113]. The biocatalytic efficacy of microbial consortia has been especially noticeable in studies on the bioconversion of coal to methane [114].

### 4.2. Bioremediation Potential of Arbuscular Mycorrhizal Fungi

Bioremediation strategies for soils affected by coal mining activities primarily aim to establish pioneer vegetation, because plant root systems stabilize degraded soils by controlling soil erosion and restoring soil fertility. The introduction of plant cover to mining areas can be facilitated by beneficial soil microorganisms, including plant-growth-promoting (PGP) bacteria and arbuscular mycorrhizal fungi (AMF). These essential microbial “biocatalysts” provide mutualistic support to sustain plant growth in degraded lands by enhancing the ability of plants to resist predominant conditions and by increasing plant performance through the biotransformation of pollutants to less toxic forms [49,115]. AMF, due to their vast mycelial network, can colonize plants and effectively extract nutrients and water from soil [116]. In addition, AMF enhance the root microbial community structure and contribute to the capture of macro- and micronutrients by plants [117]. Very recent studies by Sekhohola-Dlamini et al. and Widhayasa et al. showed that the period of post-coal-mining reclamation, vegetation, and soil physicochemical properties are profoundly determined by the soil AMF [117,118].

Another beneficial aspect of AMF in ecosystems is the facilitation of carbon conservation in coalfield soils. For example, in a study by Wang et al. [119], increased AMF inoculation significantly enhanced carbon sequestration, respiration, and photosynthesis in many plant species (wild cherry, cerasus humilis, shiny leaf yellow horn, and apricot). In the same study, the AMF promoted plant growth, significantly increasing the leaf area, chlorophyll content, and Q_10_ value.

Taheri et al. [120] tested the potential for AMF to mediate plant adaptation to mine soil conditions and found that the plants (common grass and forb) with fungal communities (dominated by *Paraglomus occultum*, but also harbored an undescribed *Entropospora* species and *Glomus mosseae* derived from mine soil) grew larger, regardless of the soil type in which they were grown. The authors suggest that microbial communities collected from harsh environments can be the best group to draw upon for the fungal inoculation of nursery plants destined to be planted in reclaimed areas. A field experiment was conducted by Bi et al. [121] to study the ecological effects of AMF (*Funneliformis mosseae* and *Rhizophagus intraradices*) on the growth of *Amygdalus pedunculata* Pall. and their root development in coal-mine-subsided areas. The results showed that AMF increased the quantity of microorganisms in the rhizosphere as well as soil quality compared with the non-inoculated treatment.

Salim et al. observed that the AMF population shows a tendency to increase along with the increasing revegetation age classes. In one of their studies, the eight-year revegetation age classes had the highest average number of spores [122]. Another study by the same research group [123] showed that the increase in revegetation age led to an increase in the number of AMF populations, with *Glomus* sp. and *Acaulospora* sp. being the dominant AMF representatives in every land revegetation age.

The combined inoculation of AMF *(Glomus mosseae*) and phosphate solubilizing bacteria (*Pantoesstewarti*) significantly enhanced the soil quality and ecological efficiency of the coal mining waste, and also improved the plant biomass of *Medicago sativa* L. [124]. These results imply that bacteria and AMF together play an essential role in phytate mineralization and subsequent transfer to the host plant.

Of particular interest are the approaches involving biofertilizers (*Rhizobium* sp., *Azotobacter* sp.), effluent treatment plant sludge, and mycorrhizal fungi (*Glomus* sp. and *Gigaspora* sp. isolated from plants growing near mine spoil dumpsites) along with suitable plant species. Inoculating such biofertilizers allowed Juwarkar et al. to reduce heavy metals and improve the rhizosphere microbiological characteristics for plant growth [125].

Fungcoal, exploiting fungi–plant mutualism, was developed in South Africa as a viable and alternative strategy for rehabilitating coal discard dumps and opencast spoils [126]. In short, Fungcoal is composed of mutualistic networks between (a) C4 grasses (*Eragrostis tef*, *Cynodon dactylon*, and *Pennisetum clandestinum*, (b) AMF inoculates (*Paraglomus occultum*, *Glomus clarum*, *Glomus mossea*, and *Gigaspora gigantea*), and (c) coal-degrading fungi (*Neosartorya fischeri* ECCN 84 and/or *Aspergillus* ECCN 84). Fungcoal was created as a strategy to address numerous issues, including (1) biodegradation of carbon pollutants, (2) biogeneration of technosol with humified organic matter, (3) promotion of mutualism between plants and microbes, and (4) activation of relevant rhizosphere microbes, etc. [117]. Based on in situ studies, the authors postulated that soil fertility can be improved de novo through a microbial consortium, so-called ‘humifiers’, and that combinations of specific biocatalysts act synergistically in maintaining soil dynamics.

Mutualistic interaction between plant roots and nonmycorrhizal fungi can also aid in the growth of plants. For example, in a study conducted by Igbinigie et al. [127], a phyto-bioconversion of hard coal involving plants and free-living fungi (*Aspergillus* spp., *Ulocladium* sp., *Alternaria* sp., and *Penicillium* sp.) occurring in the rhizosphere facilitated the growth of *Cynodon dactylon* (Bermuda grass) in the coal dump.

### 4.3. Rhizosphere Microbial Community as a Bioindicator of Soil Restoration in Coal Mining Sites

The community structure and composition of soil microorganisms undergo considerable taxonomic and functional changes during remediation and restoration in disturbed coal mining ecosystems. In the last two decades, the interaction between the rhizosphere microbial communities and their host plants in mining-affected environments and their responses to various factors have received considerable scientific attention. The microbiological nature of the rhizosphere presents essential information regarding the screening and management of plant species for degraded land revegetation. The studies summarized in Table 4 illustrate the idea that assessing microbial diversity in coal mine-affected soils may be a sensitive indicator of ecological stress and the restoration processes. The accompanying comprehensive analyses conducted by the authors reveal the dynamic nature and complexity of the soil microbial composition in the field of vegetation restoration in mining areas (Table 4).

### 4.4. Bioremediation of Heavy Metals and Selenium in Coal Mining Areas

Heavy metal pollution by industrial sources and anthropogenic activities poses severe threats to the environment and public health because of its high toxicity, non-biodegradability, and bioaccumulation. Coal mining is a prominent global geogenic and anthropogenic source of heavy metal pollution through acid mine drainages (AMD) [145,146]. The AMD from abandoned coal mines is a serious problem faced by many countries and may remain hazardous for decades or even centuries after mine closure, persistently shaping unfavorable environmental scenarios. To date, research on the impact, assessment, and management of AMD has received extensive attention worldwide, focusing on sustainable remediation strategies. Significant efforts have been devoted to reducing heavy metal loads and enhancing microbial activities [147]. Among the biotic approaches, autochthonous microorganisms are one of the best options for the decommissioning of AMD stressors [148,149].

Sulfate-reducing bacteria (SRB), including *Desulfosporosinus*, *Desulfitobacterium*, and some members of *Firmicutes* and *Actinobacteria*, attenuating toxic metal/metalloid concentrations, have been repeatedly isolated from mining sites [150,151]. AMD treatment by SRB reduces sulfates to hydrogen sulfide, which binds with metals, thus removing them from the solution. In addition, SRB generate alkalinity, contributing to the neutralization of AMD acidity [152]. In a study by Luptakova et al., a mixed SRB culture of *Desulfovibrio* and *Desulfotomaculum* effectively removed Cu^2+^ from model solutions after 5–6 days using one batch reactor [153]. Dong et al. showed that SRB combined with coal gangue achieved high treatment efficiency in repairing AMD, i.e., it achieved the highest removal percentages of chemical oxygen, SO_4_^2−^, Fe^2+^, and Mn^2+^ [154]. SRB isolated by Ma et al. [155] from loess polluted by coal AMD exhibited almost 100% immobilization of Fe^2+^, Cd^2+^, and Zn^2+^ by hydrogen sulfide precipitation after 18 days.

The bioremediation potential of indigenous heavy metal-tolerant bacteria isolated from a rat-hole coal mine environment was well documented by Shylla et al. [156]. In their study, three isolates (*Serratia marcescens* KH-CC, *Bacillus siamensis* KH-12A, and *Bacillus altitudinis* KH-16F) out of twelve exhibited a high maximum tolerable concentration (MTC) against Pb (1400 ppm), Mn (830 ppm), and Fe (500 ppm). *Serratia marcescens* exhibited the highest Mn and Pb remediation, with 72.5 and 83% removal capacities, respectively. Ka-ot et al. [157] discovered that two bacterial isolates, *Bacillus subtilis* subsp. *inaquosorum* SK22 and *Bacillus cereus* SK44, isolated from rat-hole coal mines showed resistance to 100 mg/L of Fe and 1 mg/L of both Cd and Cr compared with the control strains of *Bacillus subtilis* MTCC 441 and *Bacillus cereus* MTCC 430. The same authors, in another study [158], aimed to profile the native bacterial isolates from a rat-hole coal mine for their bioprospection as bioremediating agents, and found that the minimum inhibitory concentration and maximum bactericidal concentration of Cd^2+^, Fe^2+^, and Cr^6+^ against *Enterobacter huaxiensis* KHED8 were 4000, 4096, and 256 mg/L, respectively. Furthermore, *E. huaxiensis* KHED8 was able to remove 89%, 90%, and 82.45% of Fe^2+^, Cd^2+^, and Cr^6+^, respectively. Previously, Zheng et al. [159] identified a total of 23 highly sensitive genera (*Actinobacteria*, *Acidobacteria*, *Candidate* division WS3, *Chloroflexi*, *Gemmatimonadetes*, *Proteobacteria*, and *Thermotogae*) and 16 highly resistant genera (*Bacteroidetes* and *Proteobacteria*) to Cd^2+^ and Hg^2+^ in coal-mine-affected agricultural soil.

Some bacterial populations can thrive under coal-overburdened strata’s mineral-depleted and highly toxic heavy metal conditions. Singh et al. [160] enumerated the bacterial diversity of active opencast coal mining sites stratum-wise and found that the bacterial isolates belonging to *Firmicutes*, *Actinobacteria*, and *Proteobacteria* exhibited high tolerance (5 to 12 mM) to heavy metals (Ni^2+^, Cu^2+^, Cr^6+^, As^3+^, and Cd^2+^) and could be promising agents for the bioremediation of contaminated sites. *Enterobacter* spp., *Klebsiella* spp., and *Acinetobacter gyllenbergii* were selected by Gandhi et al. [161] based on their high level of heavy metal resistance (Cd^2+^, Pb^2+^, Fe^2+^, Mn^2+^, and Cu^2+^) and their biochemical characterization. Interestingly, a high degree of metal resistance was associated with multiple-antibiotic resistance of these isolates.

Micromycetes can also precipitate metals as insoluble oxalates, participating in metal removal from geochemical cycling. Thermophilic/thermotolerant micromycete cultures of *Aspergillus* spp. Isolated from coal seam spoil are capable of binding Cu^2+^ as low-solubility crystalline moolooite [162]. The Ni^2+^ and Cd^2+^ resistance of fungi from coal mining environments has also been described elsewhere [163].

As mentioned, microbial consortia can exhibit excellent sequestration of various multi-component toxic heavy metal mixtures in AMD. Such consortia of bacteria indigenous to coal AMD that tolerate elevated concentrations of heavy metal mixtures can be specifically designed in order to develop targeted bioremediation strategies for alleviating heavy metal toxicity in situ [164]. For example, the study by Oyetibo et al. revealed that a consortium of seven autochthonous bacterial taxa (ϒ-Proteobacteria: groups of *Acinetobacter pittii*, *Enterobacteriaceae*, *Pseudomonas citronellolis*, and unclassified FWNZ species, and Bacilli: groups of *Sporosarcina koreensis*, *Bacillus cereus group*, and *Exiguobacterium aurantiacum*) exhibited excellent urease activities (≥253 μmol urea min^−1^) with subsequent stemming of acidic pH to >8.2 and sequestration of toxic metals (~100% efficiency) as precipitates (15.6 ± 0.92 mg ml^−1^). Bacterial ureases hydrolyze urea into ammonia and carbamate, which subsequently release ammonia and carbonic acid that can stem acidic and toxic metal impacts [165]. Naghavi et al. reported that *Acidithiobacillus ferrooxidans* isolated from coal mining, when added to Cu^2+^ coal mining samples, showed synergistic effects toward natural ferrous oxidizing microorganisms, manifested as up to a 46.7% increase in Cu^2+^ extraction [166].

A number of bacterial communities with good heavy metal tolerance and bioremediation potential in extreme environments have been discovered and characterized through high-throughput sequencing technology. According to Liu et al., metal-tolerant *Proteobacteria* and *Actinobacteria* are predominant at the phylum level in mining area soils [167]. The study by Ma et al. [168] indicated that acidic coal gangue was relatively rich with SRB, containing six genera: *Desulfosporosinus*, *Desulfovibrio*, *Desulfotomaculum*, *Desulfobulbus*, *Desulfitobacterium*, and *Desulfurella*.

Oxidation states influence the solubility and bioavailability of another important pollutant, selenium. One Se^6+^-reducing bacterium, *Enterobacter hormaechei*, and four Se^4+^-reducing bacteria, *Klebsiella pneumoniae*, *Pseudomonas fluorescens*, *Stenotrophomonas maltophilia*, and *Enterobacter amnigenus*, were isolated by Siddique et al. [169] from coal mine tailings pond sediment. The results suggested that *E. hormaechei* could remove up to 96% of the added Se^6+^ (0.92 mg L^−1^) from the effluents, with potential application in removing Se from industrial effluents.

*Bacillus paramycoides* SP3, a native strain to the leachate of coal-mine-overburden rocks, was isolated by Borah et al. [170] and investigated for its potential to produce Se nanoparticles by the biogenic reduction of selenite, one of the most toxic forms of selenium. *B. paramycoides* SP3 exhibited extremely high selenite tolerance (1000 mM). It reduced 10 mM selenite under 72 h to produce spherical monodisperse Se nanoparticles with an average size of 149.1 ± 29 nm, indicating that this strain could be utilized for the eco-friendly removal of selenite from contaminated sites with the concomitant biosynthesis of Se nanoparticles.

### 4.5. Bioremediation of Salt-Affected Soils

Soil salinization is one of the immense environmental stresses and global issues, deleteriously affecting the growth and yield of crops and thereby threatening food security. High salt concentrations in soil trigger extensive alterations in the physiology of agriculturally important plants, ultimately leading to their death. The rehabilitation and remediation of salt-affected soils have already been addressed with different technologies, including salt leaching and applying various amendments such, as gypsum and sulfuric acid [171,172].

Soil enrichment with different sources of humified organic matter and humic substances could help to alleviate the negative effect of salt accumulation on salt-sensitive crops [173]. Low-rank coal has been considered to be a superior amendment for soil quality and productivity. Low-rank coals can themselves serve as a nutritional medium for different soil microorganisms, stimulating their growth and development, and, through various metabolic mechanisms, result in a high yield of humic substances [174]. Among the microorganisms that can transform/solubilize coal to generate humified organic matter are different species of bacteria isolated from coal samples, including *Pseudomonas* sp., *Streptomyces* sp., *Rhodococcus* sp., *Bacillus* sp., and *Escherichia* sp. [175,176]. Cubillos-Hinojosa et al. reported that soil amendment with 1% coal and coal-solubilizing bacteria promoted short-term biological activity (increase in soil respiration and hydrolytic enzyme activity) associated with coal biotransformation and increased the soil cation exchange capacity [177]. Incorporating coal as a source of humic substances together with coal-solubilizing bacteria in saline–sodic soils under field conditions significantly and positively affected the chemical and biological soil properties [178]. This was reflected in decreases in the electrical conductivity, sodium adsorption ratio, and exchangeable sodium percentage, as well as in increases in microbiological activity and soil respiration [177,179]. For this reason, coal discards containing native microorganisms could be well used as an organic amendment for managing disturbed lands with the presence of salt-affected soils. However, evidence supporting the inherent chemical heterogeneity and functional diversity of coal as an amendment for salt-affected soils is scarce, and there are still uncertainties regarding the chemical mechanisms of coal as a slow-release fertilizer. Furthermore, depending on its origin and rank, coal may itself contain elevated levels of organically bound chlorides and inorganic constituents, implying a substantial risk of soil contamination [180].

## 5. Agricultural Applications of Microorganisms Native to Coal Environments

Green agriculture requires applying effective organo-mineral amendments that contain macro- and microelements and plant-growth biostimulants, which are a source of biologically active compounds. Today, low-rank coals (lignite and leonardite), as a source of humic acid, have attracted considerable interest in agriculture and environment conservation. Humic acids, as a key source of soil organic amendment, can be easily extracted and mobilized from soils through native strains of microorganisms [181]. Many of them are known as plant-growth-promoting bacteria (bio-inoculants) and represent a vital part of the healthy soil microbiome [182].

### 5.1. Production of Humic Substances through Coal Biodegradation

Humic substances are the most complex and biologically active organic matter compounds in the soil/sediments. They improve the microbial community structure and activity, regulate the availability of higher macro- and micronutrients for plant growth, and help maintain soil physicochemical properties. Furthermore, humic substances can play a considerable role in increasing plant resistance against common diseases and hostile environmental conditions.

Humic substances of low-rank coals or run-of-mine coals have properties very similar to those of soils’ humic substances. Typically, such coals contain between 25% and 85% humic acids, as compared with 1–5% in many soils/sediments [183]. This implies a novel and robust way to produce humic acids products from coal discards. It has long been observed that coal is susceptible to microbial attack, and a great number of studies have been published on this subject (Table 2 and Table 3). Among these studies, many investigated humified organics as products of coal biodegradation. However, limited evidence exists concerning the targeted production of humic acid and fulvic acid-like substances.

Microbial consortia from coal- and diesel-contaminated soil slurries appear to be more efficient as biocatalysts in degrading coal than individual strains. Such associations of bacterial strains can grow on humic acid and fulvic acid as the sole carbon source [57]. In the study by Olawale et al., the most effective coal-degrading consortia contained native strains, either *Serratia* ECCN 24b or *Exiguobacterium* ECCN 21b, or both, and these decreased the coal substrate mass by ~30% and ~10%, respectively.

Commercial coal-derived humic substances used for crop cultivation could be considered as suitable substrates for isolating microbial strains capable of stimulating plant growth. For example, bacterial and fungal genera (i.e., *Bacillus* and *Aspergillus*) isolated from raw humic substances resulted in a significant increase in lettuce biomass in hydroponic cultivations and enhanced resistance to NaCl-related abiotic stresses [184].

Coal-degrading fungi from coal-polluted sites possess the potential for bioconversion of coal to value-added products, including humic substances. Based on the accumulation of humic acid, which is a marker of successful biosolubilization, *Mucor circinelloides* (118.9 mg/L) and *Aspergillus tubingensis* (43.9 mg/L) were the most active fungi in a study reported by Nsa et al. [185]. On the other hand, *Cunninghamella bertholletiae* (67.03 mg/L), *Simplicillium subtropicum* (45.95 mg/L), *Penicillium daleae* (42.70 mg/L), and *Trichoderma koningiopsis* (42.43 mg/L) produced high amounts of fulvic acid, indicating the occurrence of depolymerization. *Penicillium ortum* MJ51 was isolated by Li et al. from lignite, and they used its cell-free filtrates to extract HA from lignite [186].

The results of the recent study by Valero et al. [187] provided further evidence that low-rank coals, alone or inoculated with native bacteria (*Bacillus mycoides*, *Acinetobacter baumannii*, and *Microbacterium* sp.), serve as a valuable amendment to improve soil reclamation processes after mining activities.

### 5.2. Coal Microorganisms with Plant-Growth-Promoting Characteristics

Plant-growth-promoting (PGP) bacteria have already gained worldwide recognition within sustainable agriculture and soil remediation: they are responsible for a broad scope of biotic activities as they positively influence plant growth, hormone balance, immunity pathways, and plant stress resistance, and improve soil nutrient availability. PGP bacteria solubilize inorganic phosphorus compounds by producing organic acids and acid phosphatases and, in return, gain root-borne carbon compounds essential for bacterial growth [188]. Therefore, coal gangue and coal discard contain phosphorus and other essential macronutrients in a form available for plant growth and development [189].

Coal-solubilizing/degrading bacteria isolated from coal slurry from discard dumps often display characteristics typical of PGP bacteria. To bring more clarity to this issue, Titilawo et al. [190] sought to establish the genetic relatedness of coal-degrading rhizosphere bacteria (*Bacillus*, *Escherichia*, *Citrobacter*, *Serratia*, *Exiguobacterium*, and *Microbacterium*) from coal discard dumps to sequences of PGP bacteria from the NCBI GenBank database. Analyses of indole and ammonium production revealed that these bacteria may have PGP characteristics.

The strains *Pseudomonas* sp. NU36 and *Acinetobacter* sp. NU25 from dump soils were characterized by Upadhyay et al. using the BIOLOG identification system that showed their high plant growth promotion effect (arising from indole-3-acetic acid (IAA) production, siderophore production, and the potential to solubilize inorganic phosphate) combined with excellent stress tolerance characteristics (high temperatures, drought, salt stress, pH, and heavy metal toxicity) [191]. Several other studies also indicated the stress tolerance characteristics of PGP bacteria. For example, Barman et al., in their recent study [192], isolated and characterized 10 bacterial strains from the coal dumping area; among them, *Bacillus toyonensis* DD1, *B. mycoides* DD2, *B. velezensis* DD9, and *B. flexus* DD10 strains demonstrated tolerance to two or more heavy metals. Additionally, these PGP bacteria could solubilize phosphate, produce IAA, produce siderophore, and show ACC deaminase activity.

An alternative to the recovery of degraded coal mining areas is revegetation with fast-growing species of legumes, which promote nutrient cycling, increase incorporation of carbon in the soil, and minimize erosion. In addition, legumes associate with symbiotic rhizobia (N-fixing bacteria), who promote increased deposition of nitrogen, reduce the soil C/N ratio, and increase soil organic matter (humus), favoring the mineralization and cycling of nutrients [193]. In a study conducted by Moura et al. [194] to evaluate the effectiveness of indigenous rhizobia isolated from coal mining areas in nodulation and their capacity to promote the growth of leguminous trees, the isolates were able to nodulate bracatinga (*Mimosa*) plants, ensuring shoot dry matter increases of 165%, and also favored nodulation and the growth of *Marica*.

Xia et al. determined whether culturable PGP isolates could be isolated from the surface of switchgrass (*Panicum virgatum* L.) from coal fields and investigated the subsequent effects of these isolates on switchgrass growth and development. A total of 307 bacterial isolates were cultured and identified into 76 strains, 36 species, and 5 phyla. Approximately 58% of bacterial strains, when reintroduced into surface-sterilized switchgrass seeds, were observed to increase the lamina length relative to the uninoculated controls [195].

The genus *Delftia* has been widely accepted as a group of PGP bacteria, although originally isolated as a free-living bacterium [196]. A new facultative chemolithoautotrophic heavy-metal-resistant *Delftia* sp. strain SR4 was isolated by Roy and Roy [197] from an open cast coal mine. It exhibited many PGP characteristics upon 48 h of incubation, including the production of IAA (23 µg mL^−1^), siderophore (55% siderophore units), ammonia (6 µmol mL^−1^), and HCN (30 ppm). Furthermore, this strain showed encouraging results on the growth of *Brassica juncea*, designating *Delftia* sp. as a versatile strain for multiple ecosystem functions.

Actinomycete strains (*Streptomyces* sp. and *Amycolatopsis* sp.) isolated from the rhizosphere of birch (*Betula pendula*) inhabiting a coal mine dump seemed to be effective in producing siderophores and antibacterial compounds, and displayed somewhat increased survival in the presence of heavy metals [198].

Plants may harbor endophytic fungi that are functionally important for their health. In a study performed by Xia et al. [199], the diversity and specificity of culturable endophytic fungal communities (the most abundant class, order, and species were *Sordariomycetes, Hypocreales*, and *Fusarium* spp., respectively) were explored in switchgrass (*Panicum virgatum* L.) growing on a reclaimed coal-mining site for around 20 years. The isolated fungi were able to enhance the heights of the shoots by about 86%, the fresh shoot weights by 69%, and the dry shoot weights by 62% after being recultivated back into the plants, demonstrating their functional features.

As already described in Chapter 4.2, arbuscular mycorrhizal fungi (AMF) are common endophytic fungi that exhibit potentially symbiotic associations with terrestrial plants. For example, inoculation with AMF *Funneliformis mosseae* significantly promoted the survival rate of sea buckthorn over 50 months while also increasing plant height after 14 (53.9%), 26 (24.2%), and 50 (16.2%) months compared with the uninoculated treatment [200]. The application of AMF (inocula of *Glomus intraradices* BEG140, *G. claroideum* BEG96, and *G. mosseae* BEG95 adapted to adverse soil conditions) together with PGP bacteria (Sinorhizobium spp. And *Azotobacter* spp.) to coal mine spoil banks could increase the growth of reed canary grass and high-biomass hemp, and compensate for reduced doses of organic amendments [201].

In spite of various technological advancements and extensive experience in employing different PGP bacteria, it is still challenging to establish an integrated bioprocess using native coal PGP bacteria on a commercial scale. Indigenous microorganisms with plant-growth-promoting characteristics should be recognized as key “bioengineers” when developing the rhizosphere in disturbed soils, as they are better adapted to local conditions and contribute to the growth of selective plants.

## 6. Coal Microalgae Hold Great Biotechnological Potential in Coal Utilization and Processing

**Figure 4:**
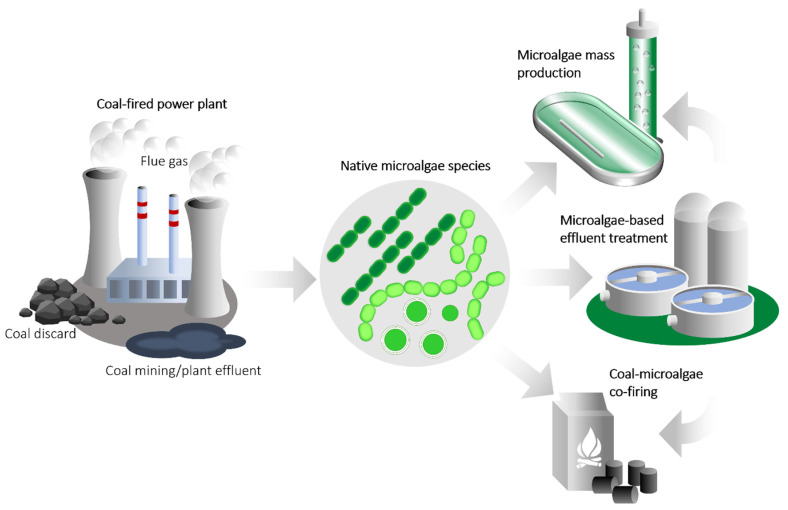
Native microalgae from the aquatic coal environment possess enormous biotechnological potential: they capture and sequester carbon to offset flue gas emissions from power plants, and the easily obtained microalgal biomass can be used for lipid/biofuel production. Coal mine drainage and power plant effluents can be decontaminated with microalgae. Finally, coal discards/fines can be burned with microalgae as a heat-efficient coal–microalgae composite. Microalgal bioremediation becomes even more attractive when the biomass cultivated in wastewater treatment systems is used as a feedstock.

Microalgae are known as bioremediation and feedstock production agents: they offer several advantages related to their high productivity, cost-efficiency, simple cultivation conditions, high stress resistance, and easy product recovery [202]. The isolation of indigenous microalgae strains and communities adapted to the coal environment is essential in both research and commercial applications for environmental sustainability and economic feasibility (Figure 4). Aquatic environments in post-coal-mining sites possess ideal conditions for the growth and productivity of microalgae. Abandoned coal mines are usually vast barren land with unlimited sunlight and carbon dioxide (CO_2_), which are the ultimate requirements for microalgal cultivation to yield great biomass [203]. Different microalgae strains could be isolated from various local coal environments, ranging from freshwater bodies to effluents generated from mining activities [204].

The main benefit of employing microalgae is associated with the bioremediation of coal-contaminated wastewater [205]. Coal mining/processing is a water-intensive industry that requires a systematic approach to treating and recycling coal effluents/drainages. For example, a typical 1000 MW coal-fired power station produces half a billion liters of metal-contaminated effluent yearly [206]. Successful wastewater treatment for the removal/biotransformation of different pollutants from coal mining areas remains challenging. However, it is worth noting that indigenous microalgae can be used as a convenient bioindicator to assess aquatic ecosystems affected by coal mining activities [207,208,209].

### 6.1. Phycoremediation

Phycoremediation is the process of employing macro and microalgae for the remediation of wastewater and effluents. This type of treatment has many advantages (self-renewing capacity, low cost, and sustainable nature) over conventional ones, which are energy-consuming, very costly, and generate a high amount of sludge [210]. Over the last few decades, algae-based systems have shown promising prospects for removing various heavy metals from AMD. Algae function as “hyper-accumulators” (when an active mechanism is involved) and “hyper-adsorbents” (through passive mechanisms), with a pronounced selectivity for different elements [211,212]. Microalgal species such as *Spirulina* sp., *Chlorella*, *Chlamydomonas*, *Scenedesmus*, *Cladophora*, and *Oscillatoria* are widely employed for heavy metal removal [212,213,214,215]. A cyanobacterium, *Nostoc* sp. KX814344, isolated by Warjri et al. [216] from a coal mine water sample showed the ability to grow at 15 ppm Cr^4+^, which is the highest Cr concentration observed in the area. In their study, Cr biosorption by *Nostoc* sp. was optimum at pH 6.0, and the biomass reached 3 μg mL^−1^. In a study by Goswami et al. [217], a cyanobacterium *Nostoc muscorum* isolated from a coal mining pit exhibited the ability to remove 66% of Zn^2+^ and 71% of Cu^2+^ within a 24 h contact time; metal binding on the cell surface was found to be the primary mode of uptake, followed by internalization.

Extremophilic indigenous coal microalgae, such as *Klebsormidium*, *Euglena*, *Mougeotia*, and *Chlamydomonas*, were repeatedly isolated from coal-generated AMD, where they were continuously exposed to very harsh chemical or physical conditions, including low pH and the presence of heavy metals [218,219,220]. In addition, the data reported by Freitas et al. [221] showed that the aquatic environment impacted by AMD could be also inhabited by *Microspora*, *Eunotia*, *Euglena*, *Mougeotia*, and *Frustulia*, accumulating huge concentrations of metals in their biomass.

Different technologies may be applied for AMD detoxification using native algae communities. For example, the oxidation pond systems containing cyanobacterial mats employed by Phillips et al. [222] showed a removal rate of up to 2.59 g Mn day^−1^ m^−2^. Bench-scale biological treatment test cells (blue–green algae, predominantly *Oscillatoria* spp./microbial mat consortium) utilized by Sheoran and Bhandari [223] were revealed to be a cost-effective treatment technique for removing SO_4_ and precipitating metals from AMD. Microcosm systems developed by Sheoran et al. using *Eunotia exigua* and *Chlamydomonas* sp. efficiently reduced the Fe content and SO_4_ level from 14 mg L^−1^ to 0.2 mg L^−1^ and from 344 mg L^−1^ to 124 mg L^−1^, respectively [45]. Finally, *Microspora quadrata*, a green filamentous algae from streams heavily contaminated by coal-generated AMD, was shown to simultaneously accumulate Pb^2+^ and Fe^2+^ ions [224].

Regarding the mechanisms of bioaccumulation, there are interesting studies conducted by Molwantwa et al. and later by Boshoff et al., where they described the pivotal role of extracellular polysaccharides. [225,226].

### 6.2. Cultivation of Microalgae with Flue Gas from Coal-Fired Power Plants

Global warming has profound implications for all aspects of ecosystems and human life. The trend of atmospheric carbon dioxide (CO_2_) emissions has been increasing over the years, and its concentration reached an average of 400 ppm, while the safe level is considered to be 350 ppm [227]. The energy power sectors are responsible for ca. 42% of CO_2_ emissions; thus, a transition toward renewable energy and alternative fuels is among the contemporary approaches to reducing CO_2_ emissions [228].

The photosynthetic process is an attractive, sustainable pathway for the biological fixation of CO_2_ by converting it into biomass as a low-carbon emission source, thus contributing to greenhouse gas reduction. Consequently, microalgae, as a globally dominant photosynthetic group, have received growing attention regarding their rapid conversion rate of CO_2_, high biomass productivity, and flexibility in the cultivation environment. It was estimated that 1 kg of dry algal biomass utilizes ~1.83 kg of CO_2_, meaning that microalgae fix waste CO_2_ ten times more efficiently than terrestrial plants [229,230]. The algal biomass can be converted into many valuable products, such as biofuel, nutritional food, and fertilizers [231].

Intensive studies focused on microalgae have been primarily conducted to develop an effective system for CO_2_ mitigation on the one hand and to increase downstream production at laboratory and industrial scales on the other. However, considerable importance should be also attached to the upstream process, such as employing and optimizing native microalgae species to expedite the acclimatization period and alleviate the in situ biological CO_2_ fixation. A significant effort toward this goal was recently made by Yahya et al. [232], who screened native microalgae species in a coal-fired power plant’s surroundings for carbon fixation ability and identified three dominant species (*Nannochloropsis* sp., *Tetraselmis* sp., and *Isochrysis* sp.). Among them, *Isochrysis* sp. was elected as the superior carbon fixer, with a fixation rate of 0.35 g CO_2_ L^−1^ day^−1^ under actual coal-fired flue gas exposure using a customized lab-scale photobioreactor. This finding was of great importance in exploring the biotechnological potential of microalgae for carbon emission mitigation from coal-based power plants.

In 2007, De Morais et al. [233] isolated *Scenedesmus obliquus* and *Chlorella kessleri* from the wastewater of a power plant and investigated their growth characteristics under different concentrations of CO_2_. The results demonstrated a high growth rate (*µ*_max_) of 0.267 day for *C. kessleri*, with a maximum biomass productivity (*P*_max_) of 0.087 g L^−1^ day^−1^ when cultivated with 6% and 12% CO_2_ and the highest maximum dry weight biomass value of 1.14 g L^−1^ with 12% CO_2_ for *S. obliquus*. Later, in a study by Radmann et al. [234], *Synechococcus nidulans* and *Chlorella vulgaris* were isolated from waste treatment ponds and compared with *Scenedesmus obliquus* and *Spirulina* sp. for CO_2_ biofixation: the results indicated that *C. vulgaris* possessed similar fixation performance to *Spirulina* sp., with a maximum daily fixation of 13.43%, when growing in reservoirs for CO_2_ biofixation from coal combustion gas.

Direct exposure to unfiltered flue gas from coal combustion is apparently challenging for microalgal communities, since they can be subjected to very high amounts of SO_x_ and NO_x_ compounds as well as heavy metals. However, mixed (“biodiverse”) microalgal communities, containing different algal genera each preadapted to high carbonate contents, can be designed and adapted to tolerate growth in as much as 100% flue gas [235]. In some cases, coal combustion wastes could even provide microalgae with minerals, which substitute the nutrients needed for their growth. Vaz et al. [236] found that *Chlorella fusca* LEB 111 and *Spirulina* sp. LEB 18 did not show significant differences in their maximum biomass concentration (ranged between 0.64 g L^−1^ and 0.58 g L^−1^) when cultivated in a natural lagoon or in a waste pond at a thermoelectric power plant, implying no significant difference in the growth support capacity between a “natural” growth medium and an “industrial” one.

Carefully designed and scaled photobioreactors for growing microalgae can possess high economic value. For instance, a novel photobioreactor (total volume of 30 m^3^) filled with *Spirulina platensis* developed by Chen et al. [237] allowed CO_2_ utilization at an annual rate of 2234 kg of CO_2_.

### 6.3. Lipid Production by Native Microalgae

Apart from the bioremediation aspects, microalgal biomass is advantageous for high-value-added product generation. Lipids extracted from the algae biomass can act as precursors to produce biodiesel. Ikenaga et al. examined the co-liquefaction of *Chlorella*, *Spirulina*, and *Littorale* with brown coal in 1-methylnaphthalene under a hydrogen atmosphere at 300–400 °C [238]. They used Fe(CO)_5_-S (at a high S/Fe ratio) and Ru_3_(CO)_12_ as catalysts and observed a high oil yield. In another study, biofuel production by *Chlamydomonas* PW95 (isolated from coal-bed methane production water) was assessed by Corredor et al. [239] using an optimal combination of culture conditions. The combination of 30 °C and 0.5 mM nitrate resulted in maximum daily biomass accumulation reaching 5.30 × 10^6^ cells/mL, high biofuel productivity (16 mg/L/d), and desirable fatty acid profiles, represented by saturated and unsaturated C16 and C18 chains. Their study may serve as a model to elicit physiological responses of microalgae to diverse culture conditions mimicking those of outdoor biofuel production.

An interesting autoflocculating microalgal strain, *Scenedesmus* sp. NC1, was isolated by Kumar et al. [240] from coal mine effluent wastewater. Its lipid characterization exhibited a complex profile (18.55% monounsaturated, 22.74% polyunsaturated, and 35.15% saturated fatty acids), designating this strain as a prospective candidate for biodiesel production. Moreover, due to its significant bioflocculation potential, *Scenedesmus* sp. NC1 can be used for better harvesting other non-flocculating microorganisms.

Abandoned coal areas can be an alternative place for the cultivation of microalgae for lipid production. Such algae samples were collected by Kumar et al. from wastewater accumulated in different coal mining areas and identified as *Spirogyra* sp. and *Oscillatoria* sp. [203]. Lipid content estimation revealed that the lipid content from algae grown in mine water was 16.3% higher than that of algae grown in river and pond water. In some cases, a double benefit could be achieved using microalgae, in which valuable biomass is produced while remediating residues for heavy metals. This was demonstrated by *Chlamydomonas acidophila* LAFIC-004 (from coal mining drainage), which is capable of growing in acidic and heavy-metal-rich mining residues [241].

### 6.4. Co-Firing of Microalgae with Coal for Power Generation

Environmental and health concerns regarding coal combustion have recently facilitated biomass utilization as a partial substitute for fossil fuels to yield high-quality coal with the desired characteristics [242]. Here, the term biomass refers to organic matter (wood, herbaceous, and aquatic biomass) generated as a result of photosynthesis and organic wastes originating from industrial, municipal, and animal materials [243]. The combustion of microalgae with fossil fuels positively affects the environment and economics of power generation. In addition, microalgae have a number of benefits, including a higher growth rate, elevated level of photosynthesis, high CO_2_ fixation efficiency, and lower requirements for environmental conditions [244].

Several studies have been conducted on the co-firing of microalgae and coal, where microalgal species have been obtained/isolated from different sources. *Scenedesmus* sp. has been reported as a promising feedstock for carbon-neutral solutions, as it offsets the CO_2_ emitted through combustion [245]. Moreover, this microalgae strain has been observed to offer higher values, i.e., 77.5 wt.% of volatile matter, 21.4 wt.% of calorific value, and low ash content (7.3 wt.%) [246]. *Scenedesmus* biomass blended with discarded ultra-fine coal has shown a prominent synergistic effect, upgrading the ignition temperature and the rate of combustion [247].

Coal–*Scenedesmus* blends, under the commercial name Coalgae^®^ 5–20% (coal and microalgae ratio at mass basis) composite, have exhibited improved combustion behavior and evolved greenhouse gases [248]. In addition, a decrease in the emissions of CO_2_, NOx, and SO_2_ from coal to Coalgae^®^ 5–20% was observed.

The following possible synergistic effects explained the co-pyrolysis of coal and *Scenedesmus* sp.: the results revealed the occurrence of three pyrolysis stages with temperature ranges of 200–400 °C, 430–650 °C, and >750 °C, and activation energies of 131–138, 72–78, and 864.5–1235 kJ/mol, respectively [249]. According to the coal pyrolysis models, three main components in microalgae (glycine, medium-chain triglyceride, and starch) were studied by Wu et al. [250]. Glycine demonstrated positive synergistic effects under a 25% mass ratio, with a higher volatile yield than the calculated value.

Subagyono et al. [251] reported the synergistic effect of mixing microalgae *Botryococcus braunii* and Victorian brown coal in co-pyrolysis reactions, dividing this process into three stages: the evaporation of water and volatile compounds (<±150 °C), active co-pyrolysis (±150–545 °C), and, finally, decomposition (±545–800 °C). The microalgae strains *Stigonematales*, *Nannochloropsis*, *Tetraselmis*, and *Chlorella* have also been employed to produce co-combustion biomass with unique combustion performance and better thermochemical properties [252,253,254]. *Stigonematales* sp. microalgae blended with coal provided an outstanding contribution to elevating volatile matter and dropping the ash content: microalgae-coal with 75%, 50%, and 25% had calorific values of 14.07 MJ/kg, 19.88 MJ/kg, and 26.42 MJ/kg, respectively [255].

An open pond microalgal culture system integrated with a coal-fired power plant seems to be a prospective setup in which the produced biomass is co-fired in the coal plant’s boiler. Geostri et al. [228] investigated the smart integration of a 500 ha microalgae *Tetraselmis suecica*-culturing facility with a large-scale coal power plant (758.6 MWe), though the produced algal biomass contributed to only around 1% of the boiler’s heat input. A fraction of the CO_2_ contained in the coal plant flue gases was used for the algal cultivation and a fraction of the low-temperature flue gas heat available was used for the biomass drying; finally, the target biomass was co-combusted in the coal plant.

## 7. Limitations

All of the above-referenced studies should facilitate an understanding of the basic concepts of coal microbiology and the applications of native microorganisms in enhancing agricultural production and environmental protection through sustainable approaches. However, there are a number of important issues that should be borne in mind when considering the various microbial groups in coal ecosystems:

1. Since every coal environment is unique in terms of nature and geology, it is difficult to formulate a set of general principles that could enhance the bio-utilization of coal universally. Furthermore, every coal source, which behaves as a part of a geomicrobial reactor, may have unique characteristics; therefore, the selection criteria of respective microbial species should be considered carefully;
2. More efforts should be made to promote better characterization of the native microorganisms, their metabolic capacities, and/or exact metabolic pathways. An understanding of the detailed mechanisms of coal biodegradation/bioconversion and their exploitation at the molecular level may be required for sustainable agricultural and environmental systems. Studies examining the metabolic and physiological characteristics of microorganisms associated with coal environments have the potential to address fundamental questions about the primary functional drivers, a key area of investigation in coal biotechnology;
3. Exploiting indigenous “microbial cocktails” native to coal may help to achieve optimized coal bioprocessing/utilization; however, this may be quite selective to a given coal environment;
4. The traditional culture-dependent techniques, despite their advantages, might have limitations in capturing and studying large variates/amounts of microorganisms from coal environments; it is thus imperative to evolve more advanced techniques to discover novel microorganisms possessing unique metabolic characteristics;
5. The underlying mechanisms of the functional roles of bacteria, fungi, and plants in coal-associated sites (abandoned mines, surface coal mines, and post-coal mining activities) and affecting environmental factors are yet to be fully explored;
6. Although recent evidence has implicated the vast potential of microalgae in coal environments, a further understanding of their ecology, adaptation mechanisms, and efficient application of these organisms could be crucial for successful bioenergy production and environmental protection.

## 8. Future Trends

1. Until now, fundamental research on coal biodegradation and bioutilization has focused mainly on the laboratory-scale screening of various methodologies and exogenous microorganisms; however, the implementation and optimization of these processes using indigenous microorganisms in full-scale outdoor systems remain attractive;
2. Many research outcomes are too preliminary to predict the details of a commercialized process. Some consideration must be given to upgrading the technology to bring these processes to regulatory issues and policy that may exert a strong influence in the future;
3. Because coal bioutilization is a complex and intricate process, efficient organisms and processes will be critical for economic competitiveness. The modern techniques of recombinant DNA technology and protein science may serve as enhanced tools for the manipulation of indigenous microorganisms;
4. Furthermore, exogenous coal-solubilizing bacteria may become part of the indigenous microbial consortia that colonize coal environments, though they may be able to naturally thrive and subsequently solubilize coal in this ecological niche.

## 9. Conclusions

The relationships of the microbial species with the coal environment are one of the critical factors for achieving increased coal degradation and utilization, as they appear to be relatively favorable microbial substrates. The heterogeneous and aromatically condensed structure of coal cannot be regarded as completely understood in terms of its bioavailability and bio-efficiency due to the complexity of coal–microbial interactions. The subsequent application of various microorganisms to benefit coal largely depends on their metabolic and functional characteristics. Therefore, establishing highly adaptive and selective microbial groups may help to determine rate-limiting steps and enhance the prospects of coal bioutilization. Furthermore, despite the lengthy research history and promising reports regarding the efficiency of indigenous microorganisms, there is still no precise prescription for successful technological innovation in a practical large-scale application.

## Figures and Tables

**Table 1:** The use of traditional techniques and the development of novel approaches have advanced the knowledge of the microbial community composition and structure residing in coal-associated environments *.

Coal Environment	Location	Study Type	Microbial Community Analysis	Microbial Community Structure	Significance	Ref.
Sediment from a brown coal basin	Sokolov brown coal basin, Czech Republic	Screening for microbial markers in sediment exposed during open-cast brown coal mining	PLFA, TLFA, and direct and cultivable microbial counts	Fungi of the genera *Penicillium*, *Verticillium*, *Cladosporium*, and *Aspergillus*, and heterotrophic bacteria of the genera *Nocardiopsis*, *Kocuria*, *Paenibacillus*, *Rothia*, *Clavibacter*, *Bacillus*, *Brevibacillus*, *Arthrobacter*, *Micrococcus*, *Microbacterium*, *Acinetobacter*, and *Pseudomonas* were isolated and characterized	Relatively high content of viable biomass and spectrum of saprotrophic fungi and heterotrophic bacteria showed that the sediment was a microbially rich geological medium in which microorganisms could survive/thrive for a long time.	[18]
Brown coal mine deposit area	Sokolov brown coal basin, Czech Republic	Assessment of the development of bacterial communities throughout the succession in the mining area	PLFA, microarray, and 16S rRNA gene-based analysis	Bacterial community composition of the 6-year-old site with no vegetation cover greatly differed from those of the older sites, especially with higher contents of *Gammaproteobacteria*, *Cyanobacteria*, and some *Alphaproteobacteria*.	Bacterial communities were especially vital during primary succession in its initial and late phases, when they dominated over soil fungi.	[19]
Stockpiles of opencast coal mines	Coal-rich Emalahleni area, South Africa	Investigation of the microbial community and enzyme activities as soil quality indicators in stockpiles of coal mines	PCR-DGGE analyses and enzyme activity determination	The bacterial OTUs spanned two phyla (*Firmicutes* and *Proteobacteria*) and four genera viz *Bacillus*, *Pseudomonas*, *Azomonas*, and *Lysinibacillus*. All fungal OTUs belonged to *Ascomycota*. Overall, the microbial community from stockpiles was impaired compared with that of the unmined site	Differences in microbial diversity and enzyme activities suggested that the soil’s biological components were highly sensitive to soil disturbance	[20]
Coal-mining-disturbed overburden	Overburden unit, USA	Assessment of the microbiological changes that occur during the maturation of surface-mining-disturbed overburden	16S rRNA gene-based analysis	Recently disturbed overburden contained an abundance of sulfur-oxidizing *Limnobacter* spp., but overburden-associated microbial communities were developed with increasing time post-disturbance	Over time, the biogeochemical weathering of disturbed overburden led to the development of microbial communities and geochemical conditions	[21]
Coal seam	Coal industry area, China	Exploration of the effects of soil extraction on microbial communities in coal seams	16S rRNA and *mcrA* gene-based analysis	Soil extraction from the shallow soil layer far away from the coal mine increased the bacterial α-diversity of the coal samples and changed the bacterial community composition	Provided basic microbial information for the subsurface microbial invasion of coal seams and helped to increase our understanding of the source of microorganisms in coal seams	[22]
Subsurface coal seams	Sydney, Surat, and Gunnedah coals, Australia	Examination of the succession and spatial partitioning of microbial ecology in coal seam environments	16S rRNA gene-based analysis	*Proteobacteria* formed a larger attached proportion (*p* < 0.0005), whereas *Firmicutes* made up a larger planktonic proportion (*p* < 0.0001). *Bacteroidetes*, *Actinobacteria*, and *Euryarchaeota* had significantly higher relative abundances in the planktonic fractions (*p* < 0.183, *p* < 0.011, and *p* < 0.0002, respectively)	Demonstrated that coal seam microbial communities undergo spatial niche partitioning during periods of succession	[12]
Spoil heaps after coal mining	Sokolov coal mining district, Czech Republic	Description of the changes in the topsoil properties of the coal mine deposit with a focus on the microbial biomass activity	PLFA and enzyme assays	Succession age affected the total and bacterial PLFA contents, followed by the soil layer and season, while for the fungal biomass content-related properties, the season was the most important	There was a general trend of increasing soil microbial biomass and the activity of enzymes in the soil during the initial phases of primary succession on spoil heaps	[23]
Coal-mining-affected soils	Subsided land due to underground coal mining, China	Exploration of the effects of different fertilizers on coal mining-affected soils and the bacterial community	16S rDNA gene-based analysis	The relative abundances of *Proteobacteria, Bacteroidetes,* and *Verrucomicrobia* increased, but the relative abundances of *Chloroflexi* and *Nitrospirae* decreased when an organic fertilizer was added	Soil reclamation via fertilization can contribute to soil recovery and bacterial community restoration over time	[24]
Coal mine sludge	Coal mine wastewater treatment plants, China	Investigation of the microbial composition and structure of industrial coal mine sludge	16S rRNA gene-based analysis	The most abundant phylum of wastewater was *Proteobacteria*, ranging from 63.64% to 96.10%, followed by *Bacteroidetes* (7.26%), *Firmicutes* (5.12%), *Nitrospira* (2.02%), *Acidobacteria* (1.31%), *Actinobacteria* (1.30%), and *Planctomycetes* (0.95%)	Most of the core genera were closely related to aromatic hydrocarbon degradation and denitrification processes, which may be helpful for wastewater management and control	[25]
Coal-mine-disturbed soils	Open-cut coal mine, Australia	Using microbial diversity to investigate the impacts of soil disturbance during open-cut mining	16S rRNA gene-based analysis	Greater species richness and evenness were revealed in rehabilitated soils as compared with non-mined soils, regardless of rehabilitation age.	Effects of inorganic fertilizer dwindled with increasing plot age, and the microbial community composition in rehabilitation sites became more equable to that in non-mined sites	[26]
Reclaimed coal mine soils	Surface coal mines, USA	Description of microbial community recovery over time in reclaimed soils	PLFA	Initial effects of surface mining resulted in total microbial biomass and diversity reductions. The total concentration of PLFA biomarkers increased after 5–14 years in soils established under plant communities	Most important phase of microbial community recovery may occur between 5 and 14 years after reclamation	[27]
Coal seam groundwater	Coal seam aquifer, Japan	The first inventory of coal seam microorganisms, along with the environmental and geochemical parameters involved	16S rRNA gene-based analysis	Bacterial genera *Acetobacterium* and *Syntrophus,* which have a symbiotic association with methanogens, were dominant; the archaeal hydrogenotrophic genus *Methanoculleus* and the methylotrophic genus *Methanolobus* were dominant	Presence of methanogens in the coal seam was suggested by methanogenic archaea and acetogenic/H_2_-generating bacteria in association with methanogens	[28]
Acid coal mine drainage	Constructed wetland system adjacent to coal mine, USA	Bacterial diversity analysis of constructed wetland that received acid drainage from an abandoned underground coal mine	16S rRNA gene-based analysis and RFLP	Approximately 40% and 35% of 16S rRNA RFLP patterns were consistent with *Acidithiobacillus ferrooxidans* and *A. thiooxidans*, respectively. Three sequences were identified as being closely associated with heterotrophic iron-oxidizing species	Dominance of the acidithiobacilli was consistent with the chemical characteristics of this site (continuous supply of reduced iron and sulfur components but with a limited amount of organic compounds)	[29]
Acid coal mine drainage	Constructed wetland system adjacent to coal mine, USA	Characterization of the microbial population present in a wetland that receives acid coal mine drainage	16S rRNA gene-based analysis	The dominant microbial species in an acid-receiving, oxic wetland were iron and sulfur oxidizers—*Acidithiobacillus thiooxidans* and *A. ferrooxidans*.	Presence of iron and sulfur oxidizers in the iron precipitate samples was consistent with the biological oxidation of iron and sulfur compounds	[30]
Interbedded coal deposit	Terrestrial core with lignite/coaly layers, New Zealand	Multidisciplinary (microbiological and geochemical) investigation of the coal deposit	16S rRNA gene-based analysis	Similar cell numbers (mean 1.2 × 10^6^ cm^−3^), high viability (4–32%), intact phospholipids (biomarkers for living *Bacteria*), and activity (sulfate reduction and DNA replication) occurred heterogeneously throughout the core	Prokaryotic populations and activity changed with lithology, depth, and substrates (formate, acetate, and oxalate)	[31]
Acid coal mine drainage	Closed coal mine, China	Investigation of mineralogical and bacterial diversity of a river affected by acid mine drainage	16S rRNA gene-based analysis	*Proteobacteria* and *Firmicutes* were the dominant phyla, and an apparent variation in *Firmicutes* species was observed in the creek affected by acid coal mine drainage	Variation in *Firmicutes* could be a biological index to diagnose the natural attenuation of acid coal mine drainage	[32]
Coal-bearing sediments	Marine subsurface sediments, Japan	Study of piezophilic microbial communities, which may include some spore formers buried in the deep and old coal-bearing sediment	16S rRNA gene-based analysis	The members of spore-forming bacteria within *Firmicutes* and *Actinobacteria* were predominantly detected in all enrichment cultures from ~1.5 to 2.4 km-deep sediment samples, followed by members of *Proteobacteria*, *Acidobacteria*, and *Bacteroidetes*. In addition, piezophilic bacteria closely related to *Virgibacillus pantothenticus* and *Bacillus subtilis* were isolated	Results underline that the deeply buried microorganisms are still alive and revivable. The continued use of cultivation-dependent approaches may lead to the discovery of other piezophilic bacteria and provide a direct means to learn more about their adaptation strategies	[33]
Coal mine drainage	Flooded coal mine shaft, Russia	Molecular analysis of the microbial community of the water from a flooded coal mine shaft	16S rRNA gene-based analysis	Most bacteria were proteobacteria of gamma classes (39.12%) and epsilon (18.65%). Among the *Gammaproteobacteria*, members of the genera *Thiovirga* (18.52%), *Thiothrix* (9.52%), and *Thiomicrorhabdus* (2.25%) were revealed	Presence of sulfur-oxidizing bacteria as the dominant group indicates that this process is responsible for the production of organic matter	[34]
Coal mine spoils	Opencast coal mine area, India	Investigation of the soil/spoil physicochemical and bacterial properties in an opencast coal mine	16S rRNA gene-based analysis	The study suggests the presence of all the bacterial entities, such as *Arthrobacter*, *Sinomonas*, *Paraburkholderia*, and *Bacillus,* to be potential biomarkers of mine spoils	Among the isolated bacterial population, *Arthrobacter* and *Sinomonas* were the most dominant entities used as important ecological indicators	[15]
Soils around coal-fire vents	Coal-fire vents, China	Investigation of bacterial and archaeal diversity in surface soils of coal-fire gas vents	RFLP and 16S rRNA gene-based analysis	The bacterial community was mainly composed of *Firmicutes*, *Proteobacteria*, *Acidobacteria*, *Bacteroidetes*, *Planctomycetes*, *Actinobacteria*, and unidentified groups. Archaeal phylotypes were the species of the phyla *Crenarchaeota* (97.9%) and *Thaumarchaeota* (2.1%)	Microbial communities were diverse and could contain a large number of novel cultivable species with the potential to assimilate materials by heterotrophic metabolism at high temperatures	[35]
Coal mining waste	Coal fire area, Russia	Study of the soil microbial community associated with the zone of underground coal combustion	16S rRNA gene-based analysis	The community was dominated by aerobic bacteria capable of growing autotrophically and obtaining energy via the oxidation of the main components of coal gases, hydrogen, and carbon monoxide	Expanded knowledge of microbial diversity, evolution, and mechanisms of adaptation to extreme coal environmental conditions	[36]
Sediment-exposed reject coal	Wetland impacted by rejected coal, USA	Recovering the novel bacterial diversity from a forested wetland impacted by rejected coal	16S rDNA gene-based analysis	Bacterial isolates were composed of *Acidiphilium* sp., *Acidobacterium capsulatum*, *Ferromicrobium acidophilium*, and *Leptospirillum ferrooxidans.* The archaeal community consisted mainly of the genus *Thermoplasma* and sequences of a novel type	Libraries also exhibited novel 16S rDNA types not retrieved from other habitats, indicating that significant diversity remains to be detected	[37]
Soils reclaimed after coal mining	Dave Johnson Coal Mine, USA	Study of the microbial community composition influenced by undisturbed and reclaimed soil	RFLP	Both undisturbed and reclaimed soil bacterial communities were found to be dominated by *Actinobacteria*; undisturbed and reclaimed soils contained *Actinobacteridae* and *Rubrobacteridae predominate*, respectively	Knowledge of diversity patterns within different soil matrices may greatly aid in determining ecological function and developing diagnostic measures of soil health	[38]
Coal mine drainage	Meitanba mine, China	Investigation of the bacterial diversity of modified (sulfur and ferrous sulfate) coal mine drainage samples	ARDRA	The compositions of the microbial community structure of the sulfur and ferrous sulfate coal samples (*Acidithiobacillus* spp. (98.42%), *Pseudomonas* spp. (1.54%), and *Legionella* spp. (0%)) were different from those of the control samples	Results showed that iron could play an essential role in the microbial community structure of coal mine drainage	[39]
Coal discard	Coal discard sites located within grassland, South Africa	Comparison of assessment parameters indicative of microbial community function and structure in rehabilitated asbestos- and coal-discard sites	PLFA and enzymatic assays	Viable microbial biomass was determined as 6080–29,851 and 8128–47,242 pmol g^−1^ dry weight for the coal and asbestos discard sites, respectively. The ranges for dehydrogenase activity in coal sites and asbestos were 24.3–339.5 μg INF g^−1^ 2 h^−1^ and 44.5–544.6 μg INF g^−1^ 2 h^−1^, respectively	Established minimum and maximum values for microbial community properties applicable to rehabilitated discard sites originating from both asbestos and coal mining	[17]
Brown coal colliery spoil	Sokolov coal mining district, Czech Republic	Study on the importance of culturability in heterotrophic bacterial population succession on a spoil of brown coal colliery substrate	Viable bacterial biomass, culturable to total cell ratio, and colony-forming curve	Four hundred and seventeen isolates were analyzed and assigned to 35 genera (23 G+ and 12 G−) and 81 species/biotypes (51 G+ and 30 G−). The most abundant genera were *Pseudomonas* (22% of total species), *Arthrobacter* (10 %), *Bacillus* (9%), and *Paenibacillus* (6%)	Heterotrophic bacterial population in the surface layer of brown colliery spoil changed with the length of time after deposition	[40]
Acid coal mine drainage waters	Streams in southeastern Ohio, USA	Investigation of the relationship of the Al, Fe, Mn, and Zn concentrations in *Klebsormidium*- dominant algal mats, water, and sediments	Compound microscope determination	The algal samples were primarily Composed of *K. rivulare*, with this taxon comprising 95–100% of the algal biomass. Other taxa found were the two unicellular algae *Euglena mutabilis* and *Chlamydomonas* sp., and the filamentous *Microspora* sp.	*Klebsormidium*-dominated algal mats may be a good indicator of the Fe concentration in water, but not of the contents of Al, Zn, or Mn	[41,42]
Acidic effluents	Abandoned mines in Northern Portugal	Study of two acidophilic algae as ecological indicators of acid mine drainage sites	Optical microscopy based on morphological features	Acidophilic algal colonization was dominated by *Euglena mutabilis* and *Klebsormidium* sp.	Spatial distribution of *E. mutabilis* can be used to qualitatively assess water quality improvements	[43]
Open-cast lignite mining lakes	Lusatia region, Germany	Determination of the taxonomically diverse algal flora of lignite mining lakes	Phytoplankton standard methods	The planktonic algal flora was generally dominated by flagellates belonging to *Chlamydomonas*, *Ochromonas*, *Chromulina*, *Cyathomonas*, *Lepocinclis*, and *Euglena mutabilis*	Many of the taxa were potentially heterotrophic or phagotrophic, enabling these organisms to augment scarce C and P supplies	[44]
Acidic lignite pit lakes	Lausitz lignite mining district, Germany	Microcosm experiments on acidity removal through controlled eutrophication	Laboratory microcosm experiments	Gradual adaptation and changes in the phytoplankton population were shown in a lake already contaminated by acid mine drainage	*Ochromonas* sp. and *Chlamydomonas* sp. were shown to tolerate a wide range of physical and chemical conditions and were present consistently in the lake	[45]
Reclaimed coal mine spoils	Brown coal mining district, Czech Republic, and lignite mining district, Germany	Comparison of soil algal communities in two contrasting chronosequences on reclaimed spoils after coal mining	Direct light and epifluorescence microscopy and “growth slide” method	A total of 122 species of algae were found in both areas. Green algae prevailed in both areas, but in the brown coal area, cyanobacteria and diatoms were also quite diverse. The total abundance of algae ranged mostly from 10^4^–10^7^ cells/g dry soil, and was one order higher in the brown coal area than in the lignite area	Sludge and compost fertilization resulted in the rapid formation of visible algal crusts dominated by *Klebsormidium crenulatum*	[46]
Acid mine drainage	Abandoned undergroundcoal mine, USA	Identification of the dominant algae in photosynthetic assemblages observed in acid mine drainage	Microscopic analyses	A diverse range of unicellular microalgae, such as *Chlorella*, *Cylindrocystis*, *Botryococcus*, and *Navicula*, and several filamentous forms identified as *Microspora*, *Cladophora*, and *Binuclearia*, were recorded	The observed high algal diversity may be related to the long duration of acid mine drainage flow at the site, which has led to the development of adapted algal communities	[47]

* OTU, operational taxonomic units; PCR-DGGE, PCR-denaturing gradient gel electrophoresis; PLFA, phospholipid fatty acid analysis; RFLP, restriction fragment length polymorphism; TLFA, total fatty acid profiles.

**Table 2:** Biodegradation of various coal sources through native consortia of bacteria: understanding the mechanisms, rates, and characterization of coal biodegradation is fundamental in the context of coal utilization/processing. This table considers only studies published since 2002 *.

Bacteria				Coal			Biodegradation					Ref.
Strains	Source of Isolation	Criteria of Selection	Study Type	Type	Origin	Composition (Proximate/Ultimate), %	Mechanism	Rate	Product/Process Characterization	Remarks	Significance	
*Bacillus mycoides* CSB25, *Microbacterium* sp. CSB3, *Acinetobacter* sp. CSB13, and *Enterobacter aerogenes* CSB10	Coal residues, coal sediment, and rhizosphere	Ability to grow in a medium with powdered coal	LRC solubilization in a solid matrix (1), and LRC biotransformation and HS production in vitro in a liquid medium (2)	Lignite	El Cerrejón opencast mine, Colombia	Humidity—28.44, A—11.12, V—47.79, FC—41.09, Q—4781 Kcal Kg^−1^. C—46.04, H—3.26, O—42.95, N—1.38, and S—0.13	BS	LRC biotransformation ranged from 25 to 37%, and HS production ranged from 127–3100 mg L^−1^	E4/E6 ratio values were 5.2 for the bio-HA and 4.8 for the chem-HA. In addition, bio-HA showed higher contents of N, C, H, and a lower content of O. IR spectra of bio-HA showed similar qualitative characteristics to those of chem-HA	Supramolecular structure of both HAs had a moderately high molecular weight caused by molecules with high aromatic condensation	Isolates can be used to exploit the LRC and produce HS	[58]
*Bacillus mycoides*, *Microbacterium* sp., and *Acinetobacter baumannii*	Coal residues, coal sediment, and rhizosphere	Selected based on [58]	Characterization of HAs obtained through the bacterial transformation of LRC	Lignite	El Cerrejón coal mine, Colombia	M—28.44, A—11.12, V—47.79, Q—4781 kcal kg^−1^, FC—41.09, and S—0.13	BS	N/A	FTIR, GC-MS, and other analyses revealed that Bio-HA had a lower degree of aromaticity, more of a hydrophilic tendency, lower O content, was enriched with nitrogenated functional groups, and aliphatic polar chains	Bio-HA generated by strains exhibited high structural similarity to each other; however, some differences were evident in the types of metabolites	Concept of supramolecular structures of the HA from LRC was established	[59]
*Pseudomonas* sp., *Bacillus* sp., *Trichoderma* sp., and *Phanerochaete* sp.	Soil associated with coal mines, water, and coal	Positive screening tests (indicated as growth on coal agar plates)	Biosolubilization measurement by determining coal weight loss	High volatile C to B bituminous coal	Coal mines (Salt Range at Dulmial Village, Tehsil Choa Saidan Shah, District Chakwal) of Pakistan	A—24.02, S—5.71%, and Q—9043 Btu/lb.	BS and BL	*Pseudomonas* sp.—25.93%, *Bacillus* sp.—36.36%, *Trichoderma* sp.—50%, and *Phanerochaete* sp.—66.67% in 30 days	UV-vis revealed an increase in the absorbance pattern; FTIR indicated alterations in the structure of coal	Presence of microorganisms and surface erosion of coal residues suggested their ability to survive in coal for a more extended period	Excellent potential for coal solubilization in coal methanogenesis	[60]
*Bacillus* sp. Y7	Weathered lignite minerals	Ability to form a brown halo of solubilized lignite	Biosolubilization of lignite on solid and liquid media	Lignite	Huolingele Minerals Administration Coalmine, China	C—41.30, H—2.70, O—17.95, N—1.04, and S—0.37	BS	More than 36.77% solubilized in 12 days	Bio-HA was similar to HA extracted by chemical processes from lignite, but had higher N/O and H/C atomic ratios than HA	Lignite solubilization correlated with an increase in pH	Conversion of LRC into value-added products, such as humic acid	[61]
Bacterial communities with *Bacillus licheniformis*-related bacteria	Leonardite sample	Isolation from the leonardite with a major source of humic acid	Coal biodegradation study showing alkali production and enzyme reactions	Leonardite	Coal mines in the Provinces of Xinjiang, Inner Mongolia, Shanxi, and Yunnan, China	pH between 2.0 and 7.5, and A between 4.58 and 40.25	BS	50% degradation of the leonardite within 21 days	FTIR revealed that the contents of C, O, and aliphatic carbon were similar in Bio-HA and C-HA	Production of Bio-HA had hormone-like bioactivity	Dissolution of leonardite to produce humic acid	[62]
Microbial community with the most abundant *Nitrobacter* genus	Surface soil containing small coal pieces	Samples contained microorganisms that had been in contact with coal	Biotransformation study of coal linked to nitrification	Sub-bituminous coal	Lithgow State Coal Mine, Australia	N/A	BT	Carbon fixed into nitrifying biomass constituted ~0.042% of the carbon in coal added to the culture	Hydrocarbons derived from coal were below the detectable limits, but apparently sufficient to sustain the microbial community	Interaction between nitrification and coal biodegradation processes was shown	Products of coal can feed fermentative and methanogenic processes	[63]
Autochthonous lignite microflora (*Firmicutes, Actinobacteria*, *Proteobacteria*, and *Bacteroidetes*)	Lignite samples	Stimulation of autochthonous microflora in lignite	Biodegradation study under conditions of acidic molasses fermentation	Lignite	Jóźwin IIB opencast mine, Poland	N/A	DC	Lignin contents in the substrate lignite and after biodegradation were equal at 75.0% and 76.1%, respectively	The lignin:cellulose ratio increased from 11 to 13 in lignite after its decay, indicating more intense cellulose biodegradation. The products of cellulose degradation were α- and β-glucose	Phenols, ketones, and certain organic compounds increased during biodegradation	Synergistic interactions between molasses-fermenting and lignite-degrading bacteria are set	[64]
*Streptomyces fulvissimus* K59	Brown coal samples	Ability to grow on coal	Screening of microorganisms to solubilize lignite and study the coal decomposition potential	Brown coal	Tur’ow Mine, Poland	N—0.40, C—55.50, H—6.36, and S—8.53	BL	Resultant concentration of biosolubilized lignite was 15 times higher as compared with that of crude coal	Pretreatment (nitric acid) caused increased N release (2–3%) with a simultaneous decrease in the C/N ratio (19–33) and a reduction in the S content (0.5 g/kg)	Plackett–Burman screening verified that biosolubilization was dependent on pretreatment, coal concentration, and C source	Allows for the recovery of complex aromatic compounds from LRC	[65]
*Fusarium oxysporum* 1101	Surface water in the area of a lignite mine	Based on preliminary studies	Study on the effect of substrate concentration on biosolubilization	Lignite	Lignite mine in Bełchatów, Poland	N/A	BL	3 times higher solubilization when the culture medium was supplemented with 5% of lignite than in the medium with 10%	FTIR showed that the relative intensity of the peaks reflected the concentration of functional groups in the solubilization products	Low-molecular-weight products were efficiently released and then polymerized for 8 days, and the content of condensed aromatics decreased	May be an alternative to chemical methods for obtaining HA and other value-added products	[66]
*Bacillus* sp. RKB 7	Coal mining soil	Ability to grow in a medium containing coal	Bacterial culture studies to evaluate the nature of coal-derived substances	Lignites	Oi-Karagay and Lenger coal beds, Kazakhstan	Oi-Karagay lignite: W—7.81, A—11.2, V—36.7, and Q—15,700. Lenger lignite: W—10, A—21.2, V—43, and Q—7300	BS/BC	24% of lignite (5% *w*/*v*) was solubilized under pH 8.2 within 14 days	UV-vis and elemental analysis indicated that the solubilization products had a lower degree of aromaticity and molecular weight	FTIR analysis revealed various functional groups in the obtained biosolubilization products	Can contribute to a deeper understanding of microbe–mineral interactions in coal environments	[67]
*Bacillus* sp. RKB 2	Coal mining soil	High ability to grow in a modified mineral medium with lignite	Study on lignite biosolubilization and characterization of its products	Lignite	Oi-Karagay coal deposit, Kazakhstan	W—7.8, A—12.0, V—35.8, C—74.1, H—4.7, S—0.1, O—19.7, and N—1.4	BS	Almost 26% of crude lignite (5% *w*/*v*) within 12 days	FTIR showed the diverse nature of the bacteria-induced humic substances. LC-MS was consistent with the types of compounds that were indicated by FTIR	Protein-like and fatty acid substances were one of the factors that triggered lignite biosolubilization	May be helpful in coalbeds for in situ bioutilization of low-rank coal	[68]
*Pseudomonas mendocina* B6-1	Coal samples	Indigenous to the coal	Experimental study on the demineralization of coal with the bacterial strain	Coals rich in inertinite group of macerals	Rajmahal Gondwana basin, India	M—2.39, A—29.89, V—34.11, and FC—31.30	DM	Reduction in the ash content (>5%) was achieved, and variable degrees of removal of Mn, Na, and Fe were noticed	Atomic absorption spectrophotometer revealed that As, Cd, Cu, Ni, Zn, Cr, Co, and Pb were removed	Elements such as Ni, Zn, Cr, and Cu maintained a robust negative correlation with the ash removal percentage	Could be an important tool for the beneficiation of coal to obtain clean fuel	[69]
*Pseudomonas mendocina* B6-1	Coal samples	Based on [69]	Demineralization of coal with the bacterial strain and characterization of its signatures	Coals rich in inertinite group of macerals	Rajmahal Gondwana basin, India	Referred to [69]	DM	Decrease in H (av. 3.3%), O (av. 18.96%), S (av. 13.23%), M (av. 11.61%), and A (av. 4.48%)	XRD revealed the reduction of the pyrite phase, and FTIR indicated shifting of the absorption peaks compared with the control	There was a shifting of most absorption peaks of the clay minerals, which was due to bacterial action	Coal beneficiation with bacteria to remove its environmentally sensitive elements	[70]
*Pseudomonas stutzeri* BHU	Formation water of a coalbed	Ability to grow in mineral salt medium with coal	Study on the coal-induced biosurfactant production	Lignite, bituminous, and anthracite	N/A	N/A	BS	When the three types of coal were used, lignite was maximally solubilized after 7 days	Hemolytic test, bacterial growth inhibition, and FTIR analysis showed the rhamnolipid nature of the biosurfactant	*P. stutzeri* produced more biosurfactant with lignite than bituminous or anthracite	May be useful in coalbeds for the in situ biotransformation of coal into methane	[71]
*Chelatococcus* strains	Formation water of a coalbed	Capable of growing on coal agar medium	Characterization of isolates to solubilize coal as a sole source of C for their growth	LRC	N/A	N/A	BS	Increase in absorbance at 450 nm and browning of the culture supernatant was observed	In a preliminary characterization, isolates provide emulsifiers (surfactants) to other bacteria that carry out coal degradation	Isolates showed higher growth in the medium with 5% coal compared with the medium without coal	Advantageous to convert sedimentary rocks into valuable products	[72]
Microbial consortia	Coal tailing water mixed with fresh cow dung	Based on a literature review	Investigation of reject coal conversion into humic substances	LRC	Jamshedpur, India	A—78	BD	N/A	FTIR spectra results showed a predominance of OH, COOH, and COO groups in HA-like compounds	Isolates were able to change and modify the macromolecular structure of reject coal	Beneficiation of rejected coal and production of value-added products	[73]
*Cupriavidus necator* SLA2, *Pseudomonas putida* SLA 32, and *Alcaligenes* sp. SLB16	Sludge enriched with coal	Ability to degrade aromatic compounds	Screening of microorganisms based on their coal-degrading activities	LRC	Untreated Indonesian coal provided by the Korea Institute of Energy Research, South Korea	M—17.36, V—43.19, A—6.75, and FC—32.88	BS	1.84% after 96 h for *C. necator* SLA2	Laccase-like activity was found in the strains when tested for RBBR dye degradation, which represented the aromatic structures present in coal	Strains were also able to increase the pH of the culture media as a response to the acidic nature of coal	Potential for the development of the biological treatment process of coal	[5]
*Citrobacter* sp. ECCN 19b, *Bacillus* sp. ECCN 41b, *Escherichia* sp. ECCN 25b, and *Bacillus* sp. ECCN 26b	Slurries of coal tailings and grass root zone on coal discard dumps	Screened for coal degradation ability in a coal medium	Study on the isolation and characterization of novel coal-degrading bacterial strains	Bituminous coal discard (1) and leonardite (2)	Emalahleni coal fields (1) and No. 2 Seam (2), South Africa	(1): M—8, A—35, V—39, FC—18, S—0.2.(2): M—4, A—40, V—49, FC—7, and S—0.2	BD	*Citrobacter* sp. ECCN 19b was able to grow and proliferate on both coals; A_600_: ~1.1 (1) and ~1.2 (2) in 20 days	Shift in pH and associated media coloration with the formation of HS, which FTIR confirmed	Preferential metabolism of alkanes from coal provided bacterial growth	Potential for the transformation of coal discard to HS	[57]

* A, ash; BC, bioconversion; BD, biodegradation; Bio-HA, (micro)biologically extracted humic acid; BL, bioliquefaction; BS, biosolubilization; BT, biotransformation; C, carbon; Chem-HA, chemically extracted humic acid; DC, decomposition; DM, demineralization; FC, fixed carbon; FTIR, Fourier-transform infrared spectroscopy; GC-MS, gas chromatography–mass spectrometry; H, hydrogen; HA, humic acid; HS, humic substances; LRC, low-rank coals; M, moisture; N, nitrogen; O, oxygen; Q, calorific value; UV-Vis, Ultraviolet–visible spectroscopy; V, volatile matter.

**Table 3:** Biodegradation of various coal sources through native strains of fungi: understanding the mechanisms, rates, and characterization of coal biodegradation is fundamental in the context of coal utilization/processing. This table considers only studies published since 2002 *.

Fungi				Coal			Biodegradation					Ref.
Strains	Source of Isolation	Criteria of Selection	Study Type	Type	Origin	Composition (Proximate/Ultimate), %	Mechanism	Rate	Product/Process Characterization	Remarks	Significance	
*Hypocrea lixii* AH	Decaying wood from mine environment	Selected based on [77]	Characterization of newly isolated lignite liquefying fungus and liquefaction products	Lignite	Fushun coal mine, China	C—74.43, H—5.26, N—1.31, S—0.49, and O—18.51	BL	As the lignite density was lower than 1.3 kg/L, its bioliquefaction reached 44.86% by weight	UV-Vis showed that the main components of bio-liquefied lignite (black liquid) were phenol derivatives, ketones, and aldehydes	GC-MS revealed 16 high-concentration compounds in black liquid, of which 11 belonged to aromatic acids or ethers	Advantageous to understand the nature of bioliquefied lignite products	[78]
*Hypocrea lixii* AH	Decaying wood from mine environment	Selected based on [78]	Quantitative measurement of coal biosolubilization	LRC	West Open Coal Mine, China	C—76.7, H—5.4, N—1.3, S—0.6, and O—16.0	BS	Highest correlation coefficient (0.995) between UV-Vis and coal bio-solubilization ratios at 513 nm	IR and UV-Vis results showed that Bio-HA and C-HA contained conjugated double bonds and aromatic ring structures	Modified UV-Vis method was developed for accurate measurement	May help the conversion kinetics by monitoring biosolubilization ratios	[79]
Fungal isolates (unidentified)	Soil sample from coal mine, decaying wood, and decaying leaves	Black liquid production corresponding to the fungus’ capability to solubilize lignite	The biosolubilzsation by estimating the liquid formation time and the weight loss of the lignite	Lignite	Fushun coal mine, China	C—76.7, H—5.4, N—1.3, S—0.6, and O—16.0	BL/BS	Nitric acid pretreatment caused 31.83% (by weight) within 11 days	Products contained aromatic acids and chain hydrocarbons, and had organic function groups of hydroxyl, cyclane, carbonyl, ether linkages, and aromatic rings	Chemical analysis indicated that side chains of lignite were important structures in the biosolubilization mechanism.	Promising coal processing technology for converting solid coal to liquid oil	[77]
*Pleurotus djamor*, *Pleurotus citrinopileatus*, and *Agpergillus* sp.	Lignite sample and rotten wood	Ability to depolymerize LRC in minimal nutrient medium	Investigation of the optimization of LRC biodepolymerization	Lignite, bituminous, and sub-bituminous coal	Neyveli, Madhuband, and Meghalya coal mines, India	Neyveli lignite: M—8.7, MM—6.1, V—46.6, FC—38.6, C—62.0, H—5.3, S—1.4, N—0.9, and O—30.4	BDP	*Pleurotus djamor* was the most efficient strain to depolymerize Neyveli lignite in comparison with the other organisms	N/A	Addition of carbon sources (sucrose, raffinose, and fructose) resulted in higher depolymerization of lignite	Coal biodepolymerization can be an alternative process for the utilization of LRC	[80]
*Trichoderma atroviride*	Lignite sample	Ability to synthesize extracellular enzymes to degrade lignite structure	Evidence for the involvement of hydrolytic and oxidative enzymes in biosolubilization	Lignite	Bergheim mine, Germany	Lithotype A	BS	Carboxylic esters and the phenolic ether bonds were cleaved by *T. atroviride* (esterases and oxidative enzymes)	Enzymes to degrade different bonds in coal were recorded with ^14^C-alkyl iodide	Lignite induced the synthesis of a specific enzyme, but no direct solubilization was involved	Direct evidence to link lignite structure degradation to enzymatic attack	[81]
*Penicillium decumbens* P6	Coal mine soil	Ability to form black droplets of lignite on plate culture	Solid-state and liquid fermentation studies to degrade coal	Lignite	Huolingele Minerals Administration Coalmine, China	C—40.32, H—4.82, S—1.25, N—4.69, and O—31.12	BD/BS	Degradation in 36 h (plate colony) and in 48 h (cell-free filtrate)	IR spectrometry and elemental analysis indicated that solubilized products displayed minor alterations to original lignite	Fulvic acid amount was high, and the molecular distribution of humic acids changed distinctively	Effective lignite degradation to produce fulvic acid	[82,83]
*Penicillium decumbens* P6	Coal mine soil	Based on the possible role of esterase from P6 isolate in depolymerizing lignite	Study to prove the roles of esterase in the enzymatic attack on lignite	Lignite	Huolingele Minerals Administration Coalmine, China	Referred to [84]	BDP	Contribution of esterase to depolymerization was about 40% in the crude supernatant	Compared with C-HA, Bio-HA had a lower percentage of aromatic carbon and ester groups, but a higher percentage of aliphatic carbon	Bio-HA promoted the growth of asparagus lettuce	Potential of esterase application in the conversion of lignite into highly bioactive compounds	[85]
*Rhizopus oryzae* AD-1	LRC sample	Isolation from coal environment and a greater capability of coal biosolubilization	The study on solubilization extension and optimization for LRC degradation	LRC	Deep coal mine in Qasam Khel, Pakistan	C—35.34, H—2.57, N—0.73, S—0.41, O—17.58, A—43.37, and Q—18,473 kJ/kg	BS/BDP	22.3% biosolubilization in the case of 0.1 mm-coal on day 5, and 36.8% at day 9 without any chemical pre-treatment	Decarboxylation, Deamination, and breaking down the side chain of the coal aromatic rings to produce a variety of aliphatic, cyclic, nitrogenous, and aromatics compounds	Coal degradation showed a substantial release of organics at 1.5% glucose and 0.5% coal loading ratio within 11 days	Can serve as a biological beneficiation of coal for alternative substances	[86]
*Hypocrea lixii* TZ1	Oxidized lignite sample	Ability to utilize coal as carbon and energy sources and solubilize solid coal particles	Biological characteristics of the fungal strain and its role in lignite bioconversion	Oxidized lignite	Fushunxi colliery, China	N/A	BC	About 23.3%	Chemical composition of lignite varied significantly when treated by strain TZ1	Complicated chemical bonds (carboxyl and hydroxyl groups) of lignite were broken by metabolites secreted by TZ1	Could play an important role in the degradation of Chinese lignite	[87]
*Penicillium chrysogenum* MW1	Core sample of sub-bituminous coal	Indigenous to the coal environment	Optimization studies on structural biodegradation of lignite	LRC	Thar coalfield, Pakistan	Huminite—77.3, liptinite—8.4, inertinite—2.6, and minerals—11.7	BD	Organics released with 0.1% glucose concentration and 1% coal after 7 days	Analytical investigations revealed the release of complex organics (polyaromatic hydrocarbons)	With increasing rank of coal, aromatic condensation increased, contributing to the unruly nature of higher-rank coals	Can help in exploiting the chemical feedstock status of coal	[88]
*Penicillium chrysogenum* MW1	Core sample of sub-bituminous coal	Indigenous to the coal environment	Coal bio-pretreatment study to make coal a suitable substrate for biological beneficiation	LRCs	Coal areas in Sindh Province, Pakistan	80 vol.% content of huminite/vitrinite	BD	After 7 days of incubation, coal particles were trapped in fungal mycelia, releasing organics	EEMS indicated the release of complex organic functionalities, and GC-MS analysis confirmed the presence of single ring aromatics, PAHs, aromatic nitrogen compounds, and aliphatics	MW1 liberated complex organic compounds from coal matrix	Can help in taking advantage of LRC reserves by converting them into alternative fuels	[74]
*Penicillium* sp. P6	Coal–soil mixture	Selected based on [82]	Characterization of lignite bio-HA and water-soluble HA	Lignite	Huolingele Minerals Administration Coalmine, China	Lignite HA: C—56.1, H—3.7, N—1.5, O—38.0, S—0.6, and A—0.1	BD	Contents of HA increased from 38.6% to 55.1% and water-soluble HA from 4.0% to 28.2%	Size-exclusion chromatography and elemental analysis revealed that the N content of Bio-HA increased by 47.36% compared with that of C-HA	After biotransformation, the molecular mass of the HA decreased, while the oxygen and nitrogen content increased	Products of lignite biodegradation had better bioactivity	[84]
*Penicillium sp.* P6	Coal–soil mixture	Based on [84]	Evaluation of the factors responsible for the increased level of N in HAs	Lignite	Huolingele Minerals Administration Coalmine, China	Lignite HA: C—56.1, H—3.7, N—1.5, O—38.0, S—0.6, and A—0.1	BD	Bio-HA in the lignite increased from 38.6% to 53.2%, depending on the ammonium sulfate concentration	CP/MAS analysis showed that the N incorporated in HA during biotransformation was in the form of free or ionized NH_2_-groups in amino acids and sugars	Amount of N incorporated in Bio-HA was related to that present in the medium	High-N-content Bio-HA has potential applications in agriculture	[89]
*Hypocrea lixii* WF8	Decaying wood around coal mines	Capable of using lignite as the sole energy source on the selective media	Biodepolymerization studies on obtaining lignite extracts (E_1_-E_5_)	Lignite	Shengli coal mine, China.	M—13.74, A—7.51, V—46.40, Q—12.68 kJ/g. C—70.84, H—5.05, and N—0.88	BDP	Maximal rate was ~35% for E_1_	Phenoxy moiety in E_1_ after biodepolimerization was significantly reduced (FTIR), while 3-phenylbutan-2-ol and 2-methyl-7-phenyl-1H-indole were produced (GC-MS)	E_1_ was recognized to be rich in HAs soluble in alkaline solution, but precipitable in acid solution	Understanding the role of ligninolytic enzymes in lignite extracts	[90]
*Trichoderma atroviride* CBS 349	Opencast coal mining area	Screened for its coal-liquefying properties	Biosolubilization study in a new type of bioreactor for solid-substrate fermentation	Lignite	From Rheinbraun AG, Germany	Lithotype A	FE	Over 40 days, 140 g of 1.5 kg of lignite held in a 25 L bioreactor was solubilized	The solubilized fraction consisted of approx. 70% HA and 30% FA-like compounds	Airmix II bioreactor (solid substrate fermentation) was effective for lignite solubilization	Devised bioreactor for lignite fermentation to produce HA and FA	[91]
*Aspergillus fumigatus* MTCC 4334	Soil samples collected from lignite mines	Capability to utilize the complex organic matter of lignite in Czepek dox medium	Investigations of lignite biosolubilization into HA by a few fungal species	Lignite	Neyveli lignite, India	M—16.7, A—11.6, V—38.7, FC—33.0.C—48.5, H—5.27, N—0.54, S—0.45, and O—28.54	BS	22.3% *w*/*w* solubilization after 45 days	Initial pH of the medium decreased with an increase in solubilization, possibly due to the production of acidic metabolites	Solubilization became constant when the lignite surface area was clogged/blocked by the cell debris	Offers an environmentally friendly and cost-effective process for HA production	[92]
*Fusarium oxysporum* LOCK 1134	Brown coal	Ability to convert solid brown coal into dark liquid droplets	Heterologous expression of laccase and its brown coal solubilization assessment	Brown coal	Bełchatów brown coal mine, Poland	C—46.23, H—5.38, O—32.40, N—0.30, S—0.84, A—14.8, and Q—18.0 Mj/kg.	BS	Amount of Bio-HA reached 1474 mg/g in culture supernatant	Elemental analysis suggested that isolate metabolized the C from coal—the amount of C decreased from 44 to 32%	*F. oxysporum* laccase was expressed in *Pichia pastoris*, which contributed to HA and FA release from liquefied coal	Obtained HA may have stimulating effects on crop growth	[93]
*Neosartorya fischeri*	Plant rhizosphere from coal dumps	Occurrence in the root zone in a coal environment	Biodegradation of hard coal in a flask and in a perfusion fixed-bed bioreactor	Hard coal	Witbank coal-producing area, South Africa	N/A	BD	Mycelia engulfment within 3 days on untreated hard coal	FTIR and GC-MS indicated oxidation of the coal surface and nitration of the condensed aromatic structures of the coal macromolecule	Biodegradation may also progress by the insertion of nitrogen groups into the condensed coal aromatic structure	May enable the development of sustainable technologies in coal mine rehabilitation	[94]
*Neosartorya fischeri* ECCN 84	Waste coal dumps	Based on [94]	Study on fungal colonization and enzyme-mediated metabolism of waste coal	LRC	Coal mines in Emalahleni (Witbank), South Africa	C—10.3 ± 2.0 mg kg^−1^, A—55.5 ± 0.3%, and Q—8–10 MJ kg^−1^	ED	Colonization of coal by the strain was associated with the formation of compact spherical pellets for 20 days	XRS of pellets showed a time-dependent decline in the weight percentage of elemental carbon and an increase in elemental oxygen	Proliferation of peroxisomes in hyphae attached to coal and increased extracellular laccase activity occurred	Supports a role of oxidative enzyme action in the biodegradation of coal	[95]

* A, ash; BC, bioconversion; BD, biodegradation; BDP, biodepolimerization; Bio-HA, (micro)biologically extracted humic acid; BL, bioliquefaction; BS, biosolubilization; C, carbon; ED, enzymatic degradation; FC, fixed carbon; FE, fermentation; FTIR, Fourier-transform infrared spectroscopy; GC-MS, gas chromatography–mass spectrometry; H, hydrogen; HA, humic acid; HS, humic substances; LRC, low-rank coals; M, moisture; N, nitrogen; O, oxygen; Q, calorific value; UV-Vis, Ultraviolet–visible spectroscopy; V, volatile matter.

**Table 4:** Culture-dependent and independent approaches used to study the active microbial community for soil development and biogeochemical cycling to ensure the sustainability of established plants in mining areas *.

Coal Area	Plant/Rhizosphere	Study Type	Microbial Community Analysis	Results	Significance	Ref.
Coal mining ecosystem	Tree species rhizosphere	Inventory of rhizosphere microbial processes of most of the inhabitant tree species of coal mining ecosystems	Microbial biomass carbon, soil enzyme activities, and basal soil respiration	Among the tree species studied, *Aegle marmelos* recorded the highest values for MBC (590 mg kg^−1^) and BSR/AMBC (0.498 mg CO_2_-C mg biomass^−1^ day^−1^)	Tree species had diverse effects on their rhizosphere, which could determine their survival and performance. Tree species could be recommended for re-vegetation	[128]
Coal mining area	*Tetraena mongolica* rhizosphere	Investigation of the effect of soil bacterial diversity near *T. mongolica* and its response to open-pit mining	Microbial community analysis via 16S rRNA profiling	Relative abundance of *Actinobacteria, Proteobacteria,* and *Gemmatimonadetes* increased, while the abundance of *Acidobacteria*, *Planctomycetes*, *Bacteroidetes*, and *Chloroflexi* decreased	Organic pollutant-degrading bacteria, such as *Sphingomonas, Gemmatimonas, Nocardioides,* and *Gaiella*, were enriched in the soil, and the carbon–nitrogen cycle was changed	[129]
Opencast mine	Brushland, forestland, grassland, and unreclaimed land	Determination of the diversity and structure of soil bacterial communities under different vegetation restorations	Microbial community analysis via 16S rRNA profiling	Vegetation restoration on the reconstructed soil in the mining area could significantly improve the OTUs, abundance (ACE and Chao1), and diversity (Shannon and Simpson) indices of the bacterial community, and the dominant phyla were *Proteobacteria*, *Actinobacteria*, and *Acidobacteria*	Since the brushland soil had better biochemical properties and higher bacterial richness and diversity, it was recommended as the optimum vegetation restoration type for soil reclamation in this area.	[130]
Coal gangue soil	Shallow (10 cm), middle (50 cm), and deep (100 cm) rhizospheres	Exploration of *C. korshinskii* in the restoration of coal gangue soil and the impact of the rhizosphere on soil micro-ecology ten years after planting	High-throughput sequencing and microbial diversity analysis using metabolome and ionomics technology	Microbial abundance increased by 8.5%, 25.0%, and 15.2% in the shallow, middle, and deep levels, respectively. S, Fe, Mn, lipids, organic acids, and oxygen compound metabolites drove *Acidthiobacillus*, *Sulfurifustis*, *Deferrobacterium*, *Pseudomonas*, and *Sphingomonas* to become dominant strains	Rhizosphere of *C. korshinskii* promoted the accumulation of remediation bacteria and accelerated the transformation and utilization of heavy metals and the process of soil remediation	[131]
Coal-discard rehabilitation sites	Discard sites vegetated with a grass seed mixture	Evaluation of the relationship between the microbial community structure, vegetation cover, and topsoil covers	PLFA	Positive association was observed between microbial biomass and the vegetation cover, organic carbon, ammonium, nitrate, and phosphorus contents	When analyzing environmental variables, including topsoil covers, the microbial community structure may be a valuable tool to assess the state of coal discard dumps under rehabilitation	[132]
Coal mine spoil heaps	Vegetation plots with various plant species	Investigation of the relationships between the plant species composition and the associated microbial properties during the process of vegetation	PLFA	Total microbial biomass in soils from the older vegetation plots was significantly higher than that in soils from the younger plots; the microbial communities consisted primarily of bacteria with the dominance of Gram-negative bacteria over Gram-positive	A strong correlation was revealed between vegetation and microbial community structure on hard coal spoil heaps	[133]
Coal mining area	Grassland, brushland, coniferous forest, and broadleaf forest	Understanding of soil microbial community functions and adaptability in mining areas	Microbial community analysis via 16S rRNA and ITS rRNA profiling	Different vegetation reconstruction modes did not affect the bacterial functional communities, but shaped different functional groups of fungi. The grassland soil was dominated by saprotrophic fungi, while symbiotrophic fungi dominated the coniferous and broadleaf forests	Findings improve the understanding of microbial ecology in reclaimed mine soil and provide a reference for the ecological restoration of fragile mining ecosystems	[134]
Coal mine spoils	Vegetated by *Medicago sativa*, *Trifolium repens*, and *Lolium perenne*	Investigation of the response of microbial communities to land reclamation	PCR-based 454 pyrosequencing	Gramineae and leguminosae herbage broadly enhanced soil geochemical characteristics and microbial diversity, representing an ideal solution for soil rehabilitation	Positive impacts of reclamation on soil microbial diversity were achieved; the most critical phase of microbial community recovery occurred between 15 and 20 years	[135]
Coal mining subsidence area	Transplanted tree species	Study of the diversity and dynamics of soil AMF in coal mining subsidence areas before and after artificially planting trees	MiSeq high-throughput sequencing	Seven genera of AMF (trees’ rhizosphere) were identified with the following abundances: *Glomus* (59.83–92.57%), *Scutellospora* (0.59–7.1%), *Diversispora* (0.59–32.73%), and others (0–0.05%). The morphological/molecular diversities in the undisturbed area were significantly higher than those in post-mining naturally restoring area	Subsidence showed positive effects on soil quality, and the trees improved the soil characteristics and increased the soil AMF diversity	[136]
Coal gangue	*Cajanus cajan* (pigeon pea) root system	Evaluation of the reclamation base of coal gangue and the analysis of the microbial diversity of the soil of reclaimed plants	MiSeq high-throughput sequencing	Dominant microflora were changed in the soil after cultivating *C. cajan*. Before cultivation, *Sulfobacteria* and *Acidobacteria* were dominant; after cultivation, *Actinobacteria*, *Acidimicubia*, *Thermoleophilia*, and *Anaerolineae* were dominant	Study proposes a reference for interactions among microorganisms in reclaimed soils for the restoration of waste coal gangue hills	[137]
Coal discard sites	Grass seed mixture	Determination of whether microbial enumeration techniques could differentiate discard sites of varying rehabilitation ages	Signature lipid biomarkers (PLFAs) and enzymatic assays	Sites with relatively higher vegetation cover and organic carbon content were positively associated with enzymatic activities and microbial biomass. Although the discard sites had different rehabilitation ages, no statistically significant differences existed	Characterization of microbial community function and structure holds potential for evaluating rehabilitation progress on mine discard sites	[138]
Coal gangue landfill	Soybean and maize rotation systems	Study of the microbial carbon metabolism function of the plant rhizosphere and non-rhizosphere soil	Biolog-EcoPlate technology	Microbial activity in the rhizosphere of plants was higher compared with that in non-rhizosphere soil, and the functional diversity of the rhizosphere microbial community was higher than those of the non-rhizosphere microbial community	Due to the changes in environmental factors in the plant growing seasons, rhizosphere and non-rhizosphere microbial composition may vary	[139]
Coal mine spoil	Succession planting (*Acacia holosericea*, *A. simsii*, and *A. harpophylla*)	Investigation of the functional diversity of the soil microbial community of a revegetated coal mine spoil exposed to the agronomic practices	Biolog-EcoPlate technology	Higher metabolic activity and functional diversity of the bacterial community in the succession planting treatment as compared with other treatments	Succession planting should be used as an important component in mine site revegetation programs	[140]
Coal mine spoil	Tree species (*A. Auriculiformis*, *Albizia lebbeck*, *Cassia siamea*, *Delonix regia*, and *Dalbergia sissoo*)	Evaluation of the effect of the tree species on the rhizosphere soil properties and identification of the key rhizosphere soil indicators that influence tree biomass	Standard protocols for the determination of total soil carbon, labile carbon, and microbial biomass carbon	Tree carbon density was significantly higher for *D. sissoo* (43.7 kg C/tree), followed by *A. auriculiformis* (39.58 kg C/tree), *D. regia* (36.3), and *C. siamea* (34.79). Total carbon was lower in all rhizosphere soils, except for *C. siamea*	Integrated carbon accumulation index and rhizosphere N could be considered indicators for carbon sequestration in reclaimed mine spoils	[141]
Coal mining soils	*Brachiaria decumbens*	Evaluation of the response of rhizobacterial communities associated with *B. decumbens* under the reclamation of coal mining soils amended with biochar	Ion torrent DNA sequencing	Application of biochar influenced the relative abundances of functional groups in the rhizosphere, including the *Sphingomonadacea*, *Rhodospirillaceae*, and *Hyphomicrobiaceae* families belonging to the *Proteobacteria*	Differences observed in the rhizobacterial community structure and abundance were related to the biochar amendment and its effect over time	[142]
Coal mining subsidence land	*Sesamum indicum*, *Glycine max*, *Medicago sativa*, *Sorghum sudanense*, and *Zea mays*	Investigation of the relationship between soil microbes and vegetation species in a reclamation area on coal mining subsidence land	Microbial community analysis via 16S rDNA	Significant growth-related dynamic changes in the microbial community structure were mainly associated with the *Proteobacteria*, *Actinobacteria*, and *Firmicutes*, which accounted for 29.69%, 13.93%, and 12.51% of the total bacterial sequences, respectively	Vegetation can improve the soil nutrient, enzyme activities, and microorganisms in the surface soil of the reclamation area and remit subsidence area with soil alkalinization	[143]
Spoil from the brown coal mine area	*Tussilago farfara*	Characterization of the rhizosphere’s effect on the bacterial community of *Tussilago farfara* colonizing the mine spoil	PLFA and NLFA	Plant roots significantly increased microbial diversity and biomass after cultivation. The rhizosphere of *Tussilago farfara* had *Bradyrhizobium japonicum*, *Rhizobium radiobacter* (bacterial nitrogen fixators), and arbuscular mycorrhizal fungi	Roots affected the microbial community and had a larger size and higher growth than the control	[144]

* NLFA, neutral lipid fraction analysis; PLFA, phospholipid fatty acid analysis.

## Data Availability

Not applicable.

## References

[1] Jones K. (2018). Ronald, E. Hester and Roy, M. Harrison (Eds): Coal in the 21st Century; Energy Needs, Chemical and Environmental Controls. Chromatographia.

[2] Zhang D., He H., Ren Y., Haider R., Urynowicz M., Fallgren P.H., Jin S., Ishtiaq Ali M., Jamal A., Adnan Sabar M. (2022). A mini review on biotransformation of coal to methane by enhancement of chemical pretreatment. Fuel.

[3] Marks C.R., Callaghan A.V., McGenity T.J. (2019). Surface and Subsurface Coal Environments: From Environmental Formation and Chemistry to Microbial Communities. Microbial Communities Utilizing Hydrocarbons and Lipids: Members, Metagenomics and Ecophysiology.

[4] Sekhohola L.M., Igbinigie E.E., Cowan A.K. (2013). Biological degradation and solubilisation of coal. Biodegradation.

[5] David Y., Baylon M.G., Pamidimarri S.D.V.N., Baritugo K.-A., Chae C.G., Kim Y.J., Kim T.W., Kim M.-S., Na J.G., Park S.J. (2017). Screening of microorganisms able to degrade low-rank coal in aerobic conditions: Potential coal biosolubilization mediators from coal to biochemicals. Biotechnol. Bioprocess Eng..

[6] Wang L., Nie Y., Tang Y.-Q., Song X.-M., Cao K., Sun L.-Z., Wang Z.-J., Wu X.-L. (2016). Diverse Bacteria with Lignin Degrading Potentials Isolated from Two Ranks of Coal. Front. Microbiol..

[7] Ishfaq H.A., Banerjee A., Qamar S., Jyothi R.K., Parhi P.K. (2021). Bio-Desulfurization of Coal Using Biotechnological Approach, Making Coal a Less Harmful Fuel. Clean Coal Technologies: Beneficiation, Utilization, Transport Phenomena and Prospective.

[8] Singh V.K., Singh R., Kumar A., Bhadouria R., Notarte K.I., Kumar A., Singh V.K., Singh P., Mishra V.K. (2021). Chapter 14—Perspectives in desulfurization of coal using microbes. Microbe Mediated Remediation of Environmental Contaminants.

[9] Ponnudurai V., Senthil Kumar P., Sankar Muthuvelu K., Velmurugan S., Subhani S., Arumugam L., Rajarathinam R. (2022). Investigation on future perspectives of ex-situ biogenic methane generation from solid waste coal and coal washery rejects. Fuel.

[10] Johnson D.B. (2003). Chemical and Microbiological Characteristics of Mineral Spoils and Drainage Waters at Abandoned Coal and Metal Mines. Water Air Soil Pollut. Focus.

[11] Méndez-García C., Peláez A.I., Mesa V., Sánchez J., Golyshina O.V., Ferrer M. (2015). Microbial diversity and metabolic networks in acid mine drainage habitats. Front. Microbiol..

[12] Vick S.H.W., Greenfield P., Pinetown K.L., Sherwood N., Gong S., Tetu S.G., Midgley D.J., Paulsen I.T. (2019). Succession Patterns and Physical Niche Partitioning in Microbial Communities from Subsurface Coal Seams. iScience.

[13] Hamidović S., Cvijović G.G., Waisi H., Životić L., Šoja S.J., Raičević V., Lalević B. (2020). Response of microbial community composition in soils affected by coal mine exploitation. Environ. Monit. Assess..

[14] Lors C., Ryngaert A., Périé F., Diels L., Damidot D. (2010). Evolution of bacterial community during bioremediation of PAHs in a coal tar contaminated soil. Chemosphere.

[15] Roy R., Mukherjee A. (2022). Physicochemical Properties and Bacterial Population of Mine Spoils in an Opencast Coal Mine Area. Proc. Natl. Acad. Sci. India Sect. B Biol. Sci..

[16] Maharana J.K., Patel A.K. (2014). Microbial community PLFA responses to ecosystem restoration in a chronosequence coal mine overburden spoil and implications of soil quality. Int. J. Curr. Microbiol. Appl. Sci..

[17] Claassens S., Jansen van Rensburg P., Liebenberg D., van Rensburg L. (2012). A Comparison of Microbial Community Function and Structure in Rehabilitated Asbestos and Coal Discard Sites. Water Air Soil Pollut..

[18] Elhottová D., Krištůfek V., Frouz J., Nováková A., Chroňáková A. (2006). Screening for microbial markers in Miocene sediment exposed during open-cast brown coal mining. Antonie Van Leeuwenhoek.

[19] Urbanová M., Kopecký J., Valášková V., Ságová-Marečková M., Elhottová D., Kyselková M., Moënne-Loccoz Y., Baldrian P. (2011). Development of bacterial community during spontaneous succession on spoil heaps after brown coal mining. FEMS Microbiol. Ecol..

[20] Ezeokoli O.T., Mashigo S.K., Paterson D.G., Bezuidenhout C.C., Adeleke R.A. (2019). Microbial community structure and relationship with physicochemical properties of soil stockpiles in selected South African opencast coal mines. Soil Sci. Plant Nutr..

[21] Poncelet D.M., Cavender N., Cutright T.J., Senko J.M. (2014). An assessment of microbial communities associated with surface mining-disturbed overburden. Environ. Monit. Assess..

[22] Li Y., Liu B., Yuan L., Xue S., Liu X., Wu Z., Chen J. (2021). Subsurface Microbial Invasion Affects the Microbial Community of Coal Seams. Energy Fuels.

[23] Baldrian P., Trögl J., Frouz J., Šnajdr J., Valášková V., Merhautová V., Cajthaml T., Herinková J. (2008). Enzyme activities and microbial biomass in topsoil layer during spontaneous succession in spoil heaps after brown coal mining. Soil Biol. Biochem..

[24] Cao Y., Zhou B., Wang X., Meng H., Zhang J., Li L., Hong J. (2020). Different fertilization treatments in coal mining-affected soils change bacterial populations and enable soil reclamation. Ann. Microbiol..

[25] Ma Q., Qu Y.-Y., Zhang X.-W., Shen W.-L., Liu Z.-Y., Wang J.-W., Zhang Z.-J., Zhou J.-T. (2015). Identification of the microbial community composition and structure of coal-mine wastewater treatment plants. Microbiol. Res..

[26] Ngugi M.R., Dennis P.G., Neldner V.J., Doley D., Fechner N., McElnea A. (2018). Open-cut mining impacts on soil abiotic and bacterial community properties as shown by restoration chronosequence. Restor. Ecol..

[27] Dangi S.R., Stahl P.D., Wick A.F., Ingram L.J., Buyer J.S. (2012). Soil Microbial Community Recovery in Reclaimed Soils on a Surface Coal Mine Site. Soil Sci. Soc. Am. J..

[28] Shimizu S., Akiyama M., Naganuma T., Fujioka M., Nako M., Ishijima Y. (2007). Molecular characterization of microbial communities in deep coal seam groundwater of northern Japan. Geobiology.

[29] Nicomrat D., Dick W.A., Dopson M., Tuovinen O.H. (2008). Bacterial phylogenetic diversity in a constructed wetland system treating acid coal mine drainage. Soil Biol. Biochem..

[30] Nicomrat D., Dick W.A., Tuovinen O.H. (2006). Assessment of the Microbial Community in a Constructed Wetland that Receives Acid Coal Mine Drainage. Microb. Ecol..

[31] Fry J.C., Horsfield B., Sykes R., Cragg B.A., Heywood C., Kim G.T., Mangelsdorf K., Mildenhall D.C., Rinna J., Vieth A. (2009). Prokaryotic Populations and Activities in an Interbedded Coal Deposit, Including a Previously Deeply Buried Section (1.6–2.3 km) Above ~150 Ma Basement Rock. Geomicrobiol. J..

[32] He J., Li W., Liu J., Chen S., Frost R.L. (2019). Investigation of mineralogical and bacteria diversity in Nanxi River affected by acid mine drainage from the closed coal mine: Implications for characterizing natural attenuation process. Spectrochim. Acta Part A Mol. Biomol. Spectrosc..

[33] Fang J., Kato C., Runko G., Nogi Y., Hori T., Li J., Morono Y., Inagaki F. (2017). Predominance of Viable Spore-Forming Piezophilic Bacteria in High-Pressure Enrichment Cultures from ~1.5 to 2.4 km-Deep Coal-Bearing Sediments below the Ocean Floor. Front. Microbiol..

[34] Kadnikov V.V., Mardanov A.V., Beletsky A.V., Antsiferov D.V., Kovalyova A.A., Karnachuk O.V., Ravin N.V. (2019). Sulfur-Oxidizing Bacteria Dominate in the Water from a Flooded Coal Mine Shaft in Kuzbass. Microbiology.

[35] Zhang T., Xu J., Zeng J., Lou K. (2013). Diversity of prokaryotes associated with soils around coal-fire gas vents in MaNasi county of Xinjiang, China. Antonie Van Leeuwenhoek.

[36] Kadnikov V.V., Mardanov A.V., Beletsky A.V., Karnachuk O.V., Ravin N.V. (2021). Metagenomic Analysis of the Microbial Community in the Underground Coal Fire Area (Kemerovo Region, Russia) Revealed Predominance of Thermophilic Members of the Phyla Deinococcus-Thermus, Aquificae, and Firmicutes. Microbiology.

[37] Brofft J.E., McArthur J.V., Shimkets L.J. (2002). Recovery of novel bacterial diversity from a forested wetland impacted by reject coal. Environ. Microbiol..

[38] Mummey D.L., Stahl P.D. (2004). Analysis of Soil Whole- and Inner-Microaggregate Bacterial Communities. Microb. Ecol..

[39] Yang Y., Ren G., Peng Z., Wang X. (2013). Bacterial Diversity Analysis of Coal Mine Drainage Enriched by Different Energy Sources. Energy Fuels.

[40] Krištůfek V., Elhottová D., Chroňáková A., Dostálková I., Picek T., Kalčík J. (2005). Growth strategy of heterotrophic bacterial population along successional sequence on spoil of brown coal colliery substrate. Folia Microbiol..

[41] Stevens A.E., McCarthy B.C., Vis M.L. (2001). Metal Content of Klebsormidium-Dominated (Chlorophyta) Algal Mats from Acid Mine Drainage Waters in Southeastern Ohio. J. Torrey Bot. Soc..

[42] Gonzalez-Curiel I., Trujillo V., Montoya-Rosales A., Rincon K., Rivas-Calderon B., deHaro-Acosta J., Marin-Luevano P., Lozano-Lopez D., Enciso-Moreno J.A., Rivas-Santiago B. (2014). 1,25-dihydroxyvitamin D3 induces LL-37 and HBD-2 production in keratinocytes from diabetic foot ulcers promoting wound healing: An in vitro model. PLoS ONE.

[43] Valente T., Gomes C.L. (2007). The role of two acidophilic algae as ecological indicators of acid mine drainage sites. J. Iber. Geol..

[44] Lessmann D., Fyson A., Nixdorf B. (2000). Phytoplankton of the extremely acidic mining lakes of Lusatia (Germany) with pH ≤ 3. Hydrobiologia.

[45] Fyson A., Nixdorf B., Kalin M. (2006). The acidic lignite pit lakes of Germany—Microcosm experiments on acidity removal through controlled eutrophication. Ecol. Eng..

[46] Lukešová A. (2001). Soil Algae in Brown Coal and Lignite Post-Mining Areas in Central Europe (Czech Republic and Germany). Restor. Ecol..

[47] Prasanna R., Ratha S.K., Rojas C., Bruns M.A. (2011). Algal diversity in flowing waters at an acidic mine drainage “barrens” in central Pennsylvania, USA. Folia Microbiol..

[48] Strc D., Mastalerz M., Dawson K., MacAlady J., Callaghan A.V., Wawrik B., Turich C., Ashby M. (2011). Biogeochemistry of microbial coal-bed methane. Annu. Rev. Earth Planet. Sci..

[49] Sekhohola L.M., Cowan A.K. (2017). Biological conversion of low-grade coal discard to a humic substance-enriched soil-like material. Int. J. Coal Sci. Technol..

[50] Davison B.H., Nicklaus D.M., Misra A., Lewis S.N., Faison B.D. (1990). Utilization of microbially solubilized coal. Appl. Biochem. Biotechnol..

[51] Haider R., Ghauri M.A., Rahim M.U. (2018). On Comparison Between Fungal and Bacterial Pretreatment of Coal for Enhanced Biogenic Methane Generation. Geomicrobiol. J..

[52] Couch G.R. (2017). Biotechnology and coal: A European perspective. Bioprocessing and Biotreatment of Coal.

[53] Sudheer P.D.V.N., David Y., Chae C.G., Kim Y.J., Baylon M.G., Baritugo K.-A., Kim T.W., Kim M.-S., Na J.G., Park S.J. (2016). Advances in the biological treatment of coal for synthetic natural gas and chemicals. Korean J. Chem. Eng..

[54] Shi C., Liu X., Zhao S., Yang Z., Lu X., Tong M. (2022). Sequential degradations of Dananhu lignites by Nocardia mangyaensis and Bacillus licheniformis. Fuel.

[55] Ghani M.J., Rajoka M.I., Akhtar K. (2015). Investigations in fungal solubilization of coal: Mechanisms and significance. Biotechnol. Bioprocess Eng..

[56] Weng C., Peng X., Han Y. (2021). Depolymerization and conversion of lignin to value-added bioproducts by microbial and enzymatic catalysis. Biotechnol. Biofuels.

[57] Olawale J.T., Edeki O.G., Cowan A.K. (2020). Bacterial degradation of coal discard and geologically weathered coal. Int. J. Coal Sci. Technol..

[58] Valero N., Gómez L., Pantoja M., Ramírez R. (2014). Production of humic substances through coal-solubilizing bacteria. Braz. J. Microbiol..

[59] Valero N.O., Gómez L.C., Melgarejo L.M. (2018). Supramolecular characterization of humic acids obtained through the bacterial transformation of a low rank coal. J. Braz. Chem. Soc..

[60] Malik A.Y., Ali M., Jamal A., Ali M.I. (2017). Isolation and Characterization of Coal Solubilizing Aerobic Microorganisms from Salt Range Coal Mines, Pakistan. Geomicrobiol. J..

[61] Jiang F., Li Z., Lv Z., Gao T., Yang J., Qin Z., Yuan H. (2013). The biosolubilization of lignite by *Bacillus* sp. Y7 and characterization of the soluble products. Fuel.

[62] Gao T.-G., Jiang F., Yang J.-S., Li B.-Z., Yuan H.-L. (2012). Biodegradation of Leonardite by an Alkali-producing bacterial community and characterization of the degraded products. Appl. Microbiol. Biotechnol..

[63] Gutierrez-Zamora M.-L., Lee M., Manefield M., Thomas T. (2015). Biotransformation of coal linked to nitrification. Int. J. Coal Geol..

[64] Detman A., Bucha M., Simoneit B.R.T., Mielecki D., Piwowarczyk C., Chojnacka A., Błaszczyk M.K., Jędrysek M.O., Marynowski L., Sikora A. (2018). Lignite biodegradation under conditions of acidic molasses fermentation. Int. J. Coal Geol..

[65] Sobolczyk-Bednarek J., Choińska-Pulit A., Łaba W. (2021). Biosolubilization of low-rank coal by the newly isolated strain Streptomyces fulvissimus K59. Fuel.

[66] Miszkiewicz H., Marchut-Mikołajczyk O., Mielcarz L., Bielecki S. (2018). Fungal biosolubilization of Polish lignite: The effect of substrate concentration. Electron. J. Pol. Agric. Universities. Ser. Agron..

[67] Akimbekov N., Digel I., Abdieva G., Ualieva P., Tastambek K. (2021). Lignite biosolubilization and bioconversion by *Bacillus* sp.: The collation of analytical data. Biofuels.

[68] Akimbekov N., Digel I., Qiao X., Tastambek K., Zhubanova A. (2020). Lignite Biosolubilization by *Bacillus* sp. RKB 2 and Characterization of its Products. Geomicrobiol. J..

[69] Singh A.L., Singh P.K., Kumar A., Yadav A., Singh M.P. (2014). Experimental Study on Demineralization of Coal with Pseudomonas Mendocina Strain B6–1 Bacteria to Obtain Clean Fuel. Energy Explor. Exploit..

[70] Singh A.L., Singh P.K., Kumar A., Singh M.P. (2015). Demineralization of Rajmahal Gondwana Coals by Bacteria: Revelations from X-ray Diffraction (XRD) and Fourier Transform Infra Red (FTIR) Studies. Energy Explor. Exploit..

[71] Singh D.N., Tripathi A.K. (2013). Coal induced production of a rhamnolipid biosurfactant by Pseudomonas stutzeri, isolated from the formation water of Jharia coalbed. Bioresour. Technol..

[72] Singh D.N., Tripathi A.K. (2011). Evaluation of the coal-degrading ability of Rhizobium and Chelatococcus strains isolated from the formation water of an Indian coal bed. J. Microbiol. Biotechnol..

[73] Saha P., Sarkar S. (2019). Microbial Degradation of Coal into a Value Added Product. Int. J. Coal Prep. Util..

[74] Haider R., Ghauri M.A., SanFilipo J.R., Jones E.J., Orem W.H., Tatu C.A., Akhtar K., Akhtar N. (2013). Fungal degradation of coal as a pretreatment for methane production. Fuel.

[75] Hofrichter M., Fakoussa R.M. (2001). Microbial degradation and modification of coal. Biopolymers.

[76] Gokcay C.F., Kolankaya N., Dilek F.B. (2001). Microbial solubilization of lignites. Fuel.

[77] Yin S., Tao X., Shi K., Tan Z. (2009). Biosolubilisation of Chinese lignite. Energy.

[78] Shi K.-Y., Tao X.-X., Yin S.-D., Du Y., Lv Z.-P. (2009). Bio-liquefaction of Fushun lignite: Characterization of newly isolated lignite liquefying fungus and liquefaction products. Procedia Earth Planet. Sci..

[79] Shi K.Y., Yin S.D., Tao X.X., Du Y., He H., Lv Z.P., Xu N. (2013). Quantitative Measurement of Coal Bio-solubilization by Ultraviolet-visible Spectroscopy. Energy Sources Part A Recovery Util. Environ. Eff..

[80] Selvi A.V., Banerjee R., Ram L.C., Singh G. (2009). Biodepolymerization studies of low rank Indian coals. World J. Microbiol. Biotechnol..

[81] Hölker U., Schmiers H., Grosse S., Winkelhöfer M., Polsakiewicz M., Ludwig S., Dohse J., Höfer M. (2002). Solubilization of low-rank coal by Trichoderma atroviride: Evidence for the involvement of hydrolytic and oxidative enzymes by using 14C-labelled lignite. J. Ind. Microbiol. Biotechnol..

[82] Yuan H.L., Yang J.S., Wang F.Q., Chen W.X. (2006). Degradation and solubilization of Chinese lignite by *Penicillium* sp. P6. Appl. Biochem. Microbiol..

[83] Yuan H., Yang J., Chen W. (2006). Production of alkaline materials, surfactants and enzymes by Penicillium decumbens strain P6 in association with lignite degradation/solubilization. Fuel.

[84] Dong L., Yuan Q., Yuan H. (2006). Changes of chemical properties of humic acids from crude and fungal transformed lignite. Fuel.

[85] Yang Y., Yang J., Li B., Wang E., Yuan H. (2018). An esterase from Penicillium decumbens P6 involved in lignite depolymerization. Fuel.

[86] Sabar M.A., Ali M.I., Fatima N., Malik A.Y., Jamal A., Farman M., Huang Z., Urynowicz M. (2019). Degradation of low rank coal by Rhizopus oryzae isolated from a Pakistani coal mine and its enhanced releases of organic substances. Fuel.

[87] Tao X.X., Chen H., Shi K.Y., Lv Z.P. (2010). Identification and biological characteristics of a newly isolated fungus Hypocrea lixii and its role in lignite bioconversion. Afr. J. Microbiol. Res..

[88] Haider R., Ghauri M.A., Jones E.J., Orem W.H., SanFilipo J.R. (2015). Structural degradation of Thar lignite using MW1 fungal isolate: Optimization studies. Int. Biodeterior. Biodegrad..

[89] Dong L., Yuan H. (2009). Nitrogen Incorporation into Lignite Humic Acids during Microbial Degradation. Geomicrobiol. J..

[90] Yao J.-H., Wei X.-Y., Xiao L., Ji H.-M., Zong Z.-M., Liu F.-J. (2015). Fractional Extraction and Biodepolymerization of Shengli Lignite. Energy Fuels.

[91] Hölker U., Höfer M. (2002). Solid substrate fermentation of lignite by the coal-solubilizing mould, Trichoderma atroviride, in a new type of bioreactor. Biotechnol. Lett..

[92] Tripathi R.C., Jain V.K., Tripathi P.S.M. (2009). Fungal Biosolubilization of Neyveli Lignite into Humic Acid. Energy Sources Part A Recovery Util. Environ. Eff..

[93] Kwiatos N., Jędrzejczak-Krzepkowska M., Strzelecki B., Bielecki S. (2018). Improvement of efficiency of brown coal biosolubilization by novel recombinant Fusarium oxysporum laccase. AMB Express.

[94] Igbinigie E.E., Aktins S., van Breugel Y., van Dyke S., Davies-Coleman M.T., Rose P.D. (2008). Fungal biodegradation of hard coal by a newly reported isolate, Neosartorya fischeri. Biotechnol. J..

[95] Sekhohola L.M., Isaacs M.L., Cowan A.K. (2014). Fungal colonization and enzyme-mediated metabolism of waste coal by Neosartorya fischeri strain ECCN 84. Biosci. Biotechnol. Biochem..

[96] Amster E. (2021). Public health impact of coal-fired power plants: A critical systematic review of the epidemiological literature. Int. J. Environ. Health Res..

[97] Hamidovic S., Teodorovic S., Lalevic B., Jovicic Petrovic J., Jovic J., Kikovic D., Raicevic V. (2016). Bioremediation Potential Assessment of Plant Growth-Promoting Autochthonous Bacteria: A Lignite Mine Case Study. Pol. J. Environ. Stud..

[98] Buta M., Blaga G., Paulette L., Păcurar I., Roșca S., Borsai O., Grecu F., Sînziana P.E., Negrușier C. (2019). Soil Reclamation of Abandoned Mine Lands by Revegetation in Northwestern Part of Transylvania: A 40-Year Retrospective Study. Sustainability.

[99] Weyer V.D., Truter W.F., Lechner A.M., Unger C.J. (2017). Surface-strip coal mine land rehabilitation planning in South Africa and Australia: Maturity and opportunities for improvement. Resour. Policy.

[100] Gerhardt K.E., Huang X.-D., Glick B.R., Greenberg B.M. (2009). Phytoremediation and rhizoremediation of organic soil contaminants: Potential and challenges. Plant Sci..

[101] Karaca O., Cameselle C., Reddy K.R. (2018). Mine tailing disposal sites: Contamination problems, remedial options and phytocaps for sustainable remediation. Rev. Environ. Sci. Bio/Technol..

[102] Karn R., Ojha N., Abbas S., Bhugra S. (2021). A review on heavy metal contamination at mining sites and remedial techniques. IOP Conf. Ser. Earth Environ. Sci..

[103] Klein J., Fakoussa R., Hölker U., Hofrichter M., Schmiers H., Sinder C., Steinbüchel A. (2001). Biotechnology of Coal. Biotechnology.

[104] Romanowska I., Strzelecki B., Bielecki S. (2015). Biosolubilization of Polish brown coal by Gordonia alkanivorans S7 and Bacillus mycoides NS1020. Fuel Processing Technol..

[105] Hazrin-Chong N.H., Marjo C.E., Das T., Rich A.M., Manefield M. (2014). Surface analysis reveals biogenic oxidation of sub-bituminous coal by Pseudomonas fluorescens. Appl. Microbiol. Biotechnol..

[106] Fakoussa R.M., Frost P.J. (1999). In vivo-decolorization of coal-derived humic acids by laccase-excreting fungus Trametes versicolor. Appl. Microbiol. Biotechnol..

[107] Ralph J.P., Catcheside D.E.A. (1997). Transformations of low rank coal by Phanerochaete chrysosporium and other wood-rot fungi. Fuel Processing Technol..

[108] Haider R. (2017). Coal degradation through fungal isolate RHC2 from Romanian brown coal sample. Energy Sources A: Recovery Util. Environ. Eff..

[109] Ghazali F.M., Rahman R.N.Z.A., Salleh A.B., Basri M. (2004). Biodegradation of hydrocarbons in soil by microbial consortium. Int. Biodeterior. Biodegrad..

[110] Patowary K., Patowary R., Kalita M.C., Deka S. (2016). Development of an Efficient Bacterial Consortium for the Potential Remediation of Hydrocarbons from Contaminated Sites. Front. Microbiol..

[111] Maka A., Srivastava V.J., Kllbane J.J., Akin C. (1989). Biological solubilization of untreated north dakota lignite by a mixed bacterial and a mixed bacterial/fungal culture. Appl. Biochem. Biotechnol..

[112] Mohanty G., Mukherji S. (2008). Biodegradation rate of diesel range n-alkanes by bacterial cultures Exiguobacterium aurantiacum and Burkholderia cepacia. Int. Biodeterior. Biodegrad..

[113] Benedek T., Máthé I., Táncsics A., Márialigeti K., Albert B., Lányi S. (2011). Intrinsic bioremediability of petroleum hydrocarbon contaminated sites in romania: Diveristy of bacterial community, catechol dioxygenase and alkane-monooxygenase genes. Bull. B Chem. Mat. Sci.

[114] Vick S.H.W., Gong S., Sestak S., Vergara T.J., Pinetown K.L., Li Z., Greenfield P., Tetu S.G., Midgley D.J., Paulsen I.T. (2019). Who eats what? Unravelling microbial conversion of coal to methane. FEMS Microbiol. Ecol..

[115] Medina A., Roldán A., Azcón R. (2010). The effectiveness of arbuscular-mycorrhizal fungi and Aspergillus niger or Phanerochaete chrysosporium treated organic amendments from olive residues upon plant growth in a semi-arid degraded soil. J. Environ. Manag..

[116] Ma Y., Tiwari J., Bauddh K. (2022). Plant-Mycorrhizal Fungi Interactions in Phytoremediation of Geogenic Contaminated Soils. Front. Microbiol..

[117] Sekhohola-Dlamini L.M., Keshinro O.M., Masudi W.L., Cowan A.K. (2022). Elaboration of a Phytoremediation Strategy for Successful and Sustainable Rehabilitation of Disturbed and Degraded Land. Minerals.

[118] Widhayasa B., Susanto D. (2019). Rhizosphere fungal community, soil physicochemical properties, understorey vegetation and their relationship during post-coal mining reclamation in East Kalimantan, Indonesia. Biodiversitas.

[119] Wang Z.-G., Bi Y.-L., Jiang B., Zhakypbek Y., Peng S.-P., Liu W.-W., Liu H. (2016). Arbuscular mycorrhizal fungi enhance soil carbon sequestration in the coalfields, northwest China. Sci. Rep..

[120] Taheri W.I., Bever J.D. (2010). Adaptation of plants and arbuscular mycorrhizal fungi to coal tailings in Indiana. Appl. Soil Ecol..

[121] Bi Y., Zhang Y., Zou H. (2018). Plant growth and their root development after inoculation of arbuscular mycorrhizal fungi in coal mine subsided areas. Int. J. Coal Sci. Technol..

[122] Salim M.A., Sri Wilarso Budi R., Setyaningsih L., Wahyudi I., Kirmi H. (2020). Root colonization by arbuscular mycorrhizal fungi (Amf) in various age classes of revegetation post-coal mine. Biodiversitas.

[123] Salim M.A., Budi S.W., Setyaningsih L., Kirmi H. (2019). Diversity of Arbuscular Mycorrhizal Fungi as Affected by Time Consequences Revegetation Age in Post Coal Mine Area at PT Berau Coal Tbk, East Kalimantan Indonesia. IOP Conf. Ser. Earth Environ. Sci..

[124] Bi Y., Xiao L., Liu R. (2019). Response of arbuscular mycorrhizal fungi and phosphorus solubilizing bacteria to remediation abandoned solid waste of coal mine. Int. J. Coal Sci. Technol..

[125] Juwarkar A.A., Jambhulkar H.P. (2008). Phytoremediation of coal mine spoil dump through integrated biotechnological approach. Bioresour. Technol..

[126] Cowan A.K., Lodewijks H.M., Sekhohola L.M., Edeki O.G. In situ bioremediation of South African coal discard dumps. Proceedings of the Mine Closure 2016: 11th International Conference on Mine Closure.

[127] Igbinigie E.E., Mutambanengwe C.C., Rose P.D. (2010). Phyto-bioconversion of hard coal in the Cynodon dactylon/coal rhizosphere. Biotechnol. J..

[128] Sinha S., Masto R.E., Ram L.C., Selvi V.A., Srivastava N.K., Tripathi R.C., George J. (2009). Rhizosphere soil microbial index of tree species in a coal mining ecosystem. Soil Biol. Biochem..

[129] Ruan M., Zhang Y., Chai T. (2020). Rhizosphere Soil Microbial Properties on Tetraena mongolica in the Arid and Semi-Arid Regions, China. Int. J. Environ. Res. Public Health.

[130] Li P., Zhang X., Hao M., Cui Y., Zhu S., Zhang Y. (2019). Effects of Vegetation Restoration on Soil Bacterial Communities, Enzyme Activities, and Nutrients of Reconstructed Soil in a Mining Area on the Loess Plateau, China. Sustainability.

[131] Bai D.-s., Wang Y.-w., Yang X., Lai J.-l., Luo X.-g. (2022). Effects of long-term (10 years) remediation of Caragana on soil enzyme activities, heavy metals, microbial diversity and metabolic spectrum of coal gangue. Ecol. Eng..

[132] Claassens S., Van Rensburg P.J.J., Van Rensburg L. (2006). Soil Microbial Community Structure of Coal Mine Discard Under Rehabilitation. Water Air Soil Pollut..

[133] Woźniak G., Markowicz A., Borymski S., Piotrowska-Seget Z., Chmura D., Besenyei L. (2015). The relationship between successional vascular plant assemblages and associated microbial communities on coal mine spoil heaps. Community Ecol..

[134] Zhao J., Ma J., Yang Y., Yu H., Zhang S., Chen F. (2021). Response of Soil Microbial Community to Vegetation Reconstruction Modes in Mining Areas of the Loess Plateau, China. Front. Microbiol..

[135] Li Y., Wen H., Chen L., Yin T. (2014). Succession of Bacterial Community Structure and Diversity in Soil along a Chronosequence of Reclamation and Re-Vegetation on Coal Mine Spoils in China. PLoS ONE.

[136] Guo Y., Chen J., Tsolmon B., He A., Guo J., Yang J., Bao Y. (2020). Effects of subsidence and transplanted trees on soil arbuscular mycorrhizal fungal diversity in a coal mining area of the Loess Plateau. Glob. Ecol. Conserv..

[137] Han S., Wang Y., Li Y., Shi K. (2021). Investigation of bacterial diversity in Cajanus cajan-planted gangue soil via high-throughput sequencing. Bioengineered.

[138] Claassens S., Riedel K.J., Van Rensburg L., Bezuidenhout J.J., Jansen van Rensburg P.J. (2006). Microbial community function and structure on coal mine discard under rehabilitation. S. Afr. J. Plant Soil.

[139] Zhang B.H., Hong J.P., Zhang Q., Jin D.S., Gao C.H. (2020). Contrast in soil microbial metabolic functional diversity to fertilization and crop rotation under rhizosphere and non-rhizosphere in the coal gangue landfill reclamation area of Loess Hills. PLoS ONE.

[140] Shrestha P., Gautam R., Ashwath N. (2019). Effects of agronomic treatments on functional diversity of soil microbial community and microbial activity in a revegetated coal mine spoil. Geoderma.

[141] Mukhopadhyay S., Masto R.E., Cerdà A., Ram L.C. (2016). Rhizosphere soil indicators for carbon sequestration in a reclaimed coal mine spoil. Catena.

[142] Rios Montes K., Pino N.J., Peñuela G.A., Mendoza A. (2020). Response of Rhizobacterial Community to Biochar Amendment in Coal Mining Soils with Brachiaria Decumbens as Pioneer Plant. Soil Sediment Contam. Int. J..

[143] Wu Z., Sun L., Li Y., Sun Q. (2020). Shifts in Vegetation-Associated Microbial Community in the Reclamation of Coal Mining Subsidence Land. Environ. Eng. Sci..

[144] Elhottová D., Krištůfek V., Malý S., Frouz J. (2009). Rhizosphere Effect of Colonizer Plant Species on the Development of Soil Microbial Community during Primary Succession on Postmining Sites. Commun. Soil Sci. Plant Anal..

[145] Zhang Y., Wu D., Wang C., Fu X., Wu G. (2020). Impact of coal power generation on the characteristics and risk of heavy metal pollution in nearby soil. Ecosyst. Health Sustain..

[146] Wang Z., Xu Y., Zhang Z., Zhang Y. (2021). Review: Acid Mine Drainage (AMD) in Abandoned Coal Mines of Shanxi, China. Water.

[147] Kothe E., Büchel G. (2014). UMBRELLA: Using MicroBes for the REgulation of heavy metaL mobiLity at ecosystem and landscape scAle. Environ. Sci. Pollut. Res..

[148] Schindler F., Merbold L., Karlsson S., Sprocati A.R., Kothe E. (2017). Seasonal change of microbial activity in microbially aided bioremediation. J. Geochem. Explor..

[149] Tamayo-Figueroa D.P., Castillo E., Brandão P.F.B. (2019). Metal and metalloid immobilization by microbiologically induced carbonates precipitation. World J. Microbiol. Biotechnol..

[150] Haferburg G., Kothe E. (2007). Microbes and metals: Interactions in the environment. J. Basic Microbiol..

[151] Florentino A.P., Weijma J., Stams A.J.M., Sánchez-Andrea I. (2015). Sulfur Reduction in Acid Rock Drainage Environments. Environ. Sci. Technol..

[152] Boonstra J., van Lier R., Janssen G., Dijkman H., Buisman C.J.N., Amils R., Ballester A. (1999). Biological treatment of acid mine drainage. Process Metallurgy.

[153] Luptakova A., Kusnierova M. (2005). Bioremediation of acid mine drainage contaminated by SRB. Hydrometallurgy.

[154] Dong Y., Di J., Yang Z., Zhang Y., Wang X., Guo X., Li Z., Jiang G. (2020). Study on the Effectiveness of Sulfate-Reducing Bacteria Combined with Coal Gangue in Repairing Acid Mine Drainage Containing Fe and Mn. Energies.

[155] Ma B.G., Hu Z.Q. (2015). Bioremediation of acid-mine drainage contaminated with acid and heavy metals in coal mine by sulfate-reducing bacteria. Legislation, Technology and Practice of Mine Land Reclamation.

[156] Shylla L., Barik S.K., Joshi S.R. (2021). Characterization and bioremediation potential of native heavy-metal tolerant bacteria isolated from rat-hole coal mine environment. Arch. Microbiol..

[157] Ka-ot A.L., Banerjee S., Haldar G., Joshi S.R. (2018). Acid and Heavy Metal Tolerant *Bacillus* sp. from Rat-Hole Coal Mines of Meghalaya, India. Proc. Natl. Acad. Sci. India Sect. B Biol. Sci..

[158] Ka-Ot A.L., Joshi S.R. (2022). Application of acid and heavy metal resistant bacteria from rat-hole coal mines in bioremediation strategy. J. Basic Microbiol..

[159] Zheng L., Li Y., Shang W., Dong X., Tang Q., Cheng H. (2019). The inhibitory effect of cadmium and/or mercury on soil enzyme activity, basal respiration, and microbial community structure in coal mine–affected agricultural soil. Ann. Microbiol..

[160] Singh K.N., Narzary D. (2021). Heavy metal tolerance of bacterial isolates associated with overburden strata of an opencast coal mine of Assam (India). Environ. Sci. Pollut. Res..

[161] Gandhi V., Priya A., Priya S., Daiya V., Kesari J., Prakash K., Kumar Jha A., Kumar K., Kumar N. (2015). Isolation and molecular characterization of bacteria to heavy metals isolated from soil samples in Bokaro Coal Mines, India. Pollution.

[162] Sokolyanskaya L.O., Ivanov M.V., Ikkert O.P., Kalinina A.E., Evseev V.A., Glukhova L.B., Karnachuk O.V. (2020). Copper Precipitation as Insoluble Oxalates by Thermotolerant Aspergillus spp. from Burning Wastes of Coal Mining. Microbiology.

[163] Castro-Silva M.A., Lima A.O.d.S., Gerchenski A.V., Jaques D.B., Rodrigues A.L., Souza P.L.d., Rörig L.R. (2003). Heavy metal resistance of microorganisms isolated from coal mining environments of Santa Catarina. Braz. J. Microbiol..

[164] Oyetibo G.O., Enahoro J.A., Ikwubuzo C.A., Ukwuoma C.S. (2021). Microbiome of highly polluted coal mine drainage from Onyeama, Nigeria, and its potential for sequestrating toxic heavy metals. Sci. Rep..

[165] Liu Y., Tang H., Lin Z., Xu P. (2015). Mechanisms of acid tolerance in bacteria and prospects in biotechnology and bioremediation. Biotechnol. Adv..

[166] Naghavi N.S., Emami Z.D., Emtiazi G. (2011). Synergistic copper extraction activity of Acidithiobacillus ferrooxidans isolated from copper coal mining areas. Asian J. Appl. Sci..

[167] Liu Y., Gao T., Wang X., Fu J., Zuo M., Yang Y., Yin Z., Wang Z., Tai X., Chang G. (2022). Effects of heavy metals on bacterial community surrounding Bijiashan mining area located in northwest China. Open Life Sci..

[168] Ma D., Liu X., Zhang M., Wang J., Hu X., Yan X., Tang C., Zhong J. (2021). Bioremediation of heavy metals pollution from acidic coal gangue with sulfate-reducing bacteria. IOP Conf. Ser. Earth Environ. Sci..

[169] Siddique T., Arocena J.M., Thring R.W., Zhang Y. (2007). Bacterial Reduction of Selenium in Coal Mine Tailings Pond Sediment. J. Environ. Qual..

[170] Borah S.N., Goswami L., Sen S., Sachan D., Sarma H., Montes M., Peralta-Videa J.R., Pakshirajan K., Narayan M. (2021). Selenite bioreduction and biosynthesis of selenium nanoparticles by Bacillus paramycoides SP3 isolated from coal mine overburden leachate. Environ. Pollut..

[171] Otlewska A., Migliore M., Dybka-Stępień K., Manfredini A., Struszczyk-Świta K., Napoli R., Białkowska A., Canfora L., Pinzari F. (2020). When Salt Meddles Between Plant, Soil, and Microorganisms. Front. Plant Sci..

[172] Munns R. (2005). Genes and salt tolerance: Bringing them together. New Phytol..

[173] Darwish T., Atallah T., El Moujabber M., Khatib N. (2005). Salinity evolution and crop response to secondary soil salinity in two agro-climatic zones in Lebanon. Agric. Water Manag..

[174] Giannouli A., Kalaitzidis S., Siavalas G., Chatziapostolou A., Christanis K., Papazisimou S., Papanicolaou C., Foscolos A. (2009). Evaluation of Greek low-rank coals as potential raw material for the production of soil amendments and organic fertilizers. Int. J. Coal Geol..

[175] Sharma A., Singh P., Kumar S., Kashyap P.L., Srivastava A.K., Chakdar H., Singh R.N., Kaushik R., Saxena A.K., Sharma A.K. (2015). Deciphering Diversity of Salt-Tolerant Bacilli from Saline Soils of Eastern Indo-gangetic Plains of India. Geomicrobiol. J..

[176] Egamberdieva D., Wirth S., Bellingrath-Kimura S.D., Mishra J., Arora N.K. (2019). Salt-Tolerant Plant Growth Promoting Rhizobacteria for Enhancing Crop Productivity of Saline Soils. Front. Microbiol..

[177] Cubillos-Hinojosa J.G., Valero N., Peralta Castilla A.d.J. (2017). Effect of a low rank coal inoculated with coal solubilizing bacteria for the rehabilitation of a saline-sodic soil in field conditions. Rev. Fac. Nac. De Agron. Medellín.

[178] Cubillos-Hinojosa J.G., Valero N.O., Melgarejo L.M. (2015). Assessment of a low rank coal inoculated with coal solubilizing bacteria as an organic amendment for a saline-sodic soil. Chem. Biol. Technol. Agric..

[179] Little K.R., Rose M.T., Jackson W.R., Cavagnaro T.R., Patti A.F. (2014). Do lignite-derived organic amendments improve early-stage pasture growth and key soil biological and physicochemical properties?. Crop Pasture Sci..

[180] Akimbekov N.S., Digel I., Tastambek K.T., Sherelkhan D.K., Jussupova D.B., Altynbay N.P. (2021). Low-Rank Coal as a Source of Humic Substances for Soil Amendment and Fertility Management. Agriculture.

[181] Verlinden G., Pycke B., Mertens J., Debersaques F., Verheyen K., Baert G., Bries J., Haesaert G. (2009). Application of Humic Substances Results in Consistent Increases in Crop Yield and Nutrient Uptake. J. Plant Nutr..

[182] Backer R., Rokem J.S., Ilangumaran G., Lamont J., Praslickova D., Ricci E., Subramanian S., Smith D.L. (2018). Plant Growth-Promoting Rhizobacteria: Context, Mechanisms of Action, and Roadmap to Commercialization of Biostimulants for Sustainable Agriculture. Front. Plant Sci..

[183] Dong L., Córdova-Kreylos A.L., Yang J., Yuan H., Scow K.M. (2009). Humic acids buffer the effects of urea on soil ammonia oxidizers and potential nitrification. Soil Biol. Biochem..

[184] Jeong H.J., Oh M.S., Rehman J.U., Yoon H.Y., Kim J.-H., Shin J., Shin S.G., Bae H., Jeon J.-R. (2021). Effects of Microbes from Coal-Related Commercial Humic Substances on Hydroponic Crop Cultivation: A Microbiological View for Agronomical Use of Humic Substances. J. Agric. Food Chem..

[185] Nsa I.Y., Akinyemi B.T., Bello-Akinosho M., Ezechukwu S.N., Bayode T.B., Igbinigie E.E., Adeleke R.A. (2022). Development of a saprophytic fungal inoculum for the biodegradation of sub-bituminous coal. SN Appl. Sci..

[186] Li S., Tan J., Wang Y., Li P., Hu D., Shi Q., Yue Y., Li F., Han Y. (2022). Extraction optimization and quality evaluation of humic acids from lignite using the cell-free filtrate of Penicillium ortum MJ51. RSC Adv..

[187] Valero N., Melgarejo L.M., Ramírez R. (2016). Effect of low-rank coal inoculated with coal solubilizing bacteria on edaphic materials used in post-coal-mining land reclamation: A greenhouse trial. Chem. Biol. Technol. Agric..

[188] Ciftci C., Tekdal D., Cetiner S., Singh V.P., Singh S., Tripathi D.K., Prasad S.M., Bhardwaj R., Chauhan D.K. (2021). 3—The importance of plant growth–promoting rhizobacteria for plant productivity. Abiotic Stress and Legumes.

[189] Wang J. (2014). Utilization of coal mine solid waste by phosphate-solubilizing bacteria and its application in reclamation. Legis. Technol. Pract. Mine Land Reclam..

[190] Titilawo Y., Masudi W.L., Olawale J.T., Sekhohola-Dlamini L.M., Cowan A.K. (2020). Coal-Degrading Bacteria Display Characteristics Typical of Plant Growth Promoting Rhizobacteria. Processes.

[191] Upadhyay N., Verma S., Pratap Singh A., Devi S., Vishwakarma K., Kumar N., Pandey A., Dubey K., Mishra R., Kumar Tripathi D. (2016). Soil ecophysiological and microbiological indices of soil health: A study of coal mining site in Sonbhadra, Uttar Pradesh. J. Soil Sci. Plant Nutr..

[192] Barman D., Dutta I., Jha D.K. (2022). Heavy metal resistant bacteria from coal dumping site with plant growth promoting potentials. Biologia.

[193] Marciano Marra L., Fonsêca Sousa Soares C.R., de Oliveira S.M., Avelar Ferreira P.A., Lima Soares B., de Fráguas Carvalho R., de Lima J.M., de Souza Moreira F.M. (2012). Biological nitrogen fixation and phosphate solubilization by bacteria isolated from tropical soils. Plant Soil.

[194] Moura G.G.D.d., Armas R.D.d., Meyer E., Giachini A.J., Rossi M.J., Soares C.R.F.S. (2016). Rhizobia Isolated from Coal Mining Areas in the Nodulation and Growth of Leguminous Trees. Rev. Bras. De Cienc. Do Solo.

[195] Xia Y., Greissworth E., Mucci C., Williams M.A., De Bolt S. (2013). Characterization of culturable bacterial endophytes of switchgrass (*Panicum virgatum* L.) and their capacity to influence plant growth. GCB Bioenergy.

[196] María A.M., Andrés I., Eugenio J., Héctor M., Susana C.-S. (2016). Revealing the biotechnological potential of *Delftia* sp. JD2 by a genomic approach. AIMS Bioeng..

[197] Roy S., Roy M. (2019). Characterization of plant growth promoting feature of a neutromesophilic, facultatively chemolithoautotrophic, sulphur oxidizing bacterium *Delftia* sp. strain SR4 isolated from coal mine spoil. Int. J. Phytoremediation.

[198] Ostash B., Gren T., Hrubskyy Y., Tistechok S., Beshley S., Baranov V., Fedorenko V. (2014). Cultivable actinomycetes from rhizosphere of birch (*Betula pendula*) growing on a coal mine dump in Silets, Ukraine. J. Basic Microbiol..

[199] Xia Y., Amna A., Opiyo S.O. (2018). The culturable endophytic fungal communities of switchgrass grown on a coal-mining site and their effects on plant growth. PLoS ONE.

[200] Zhang Y., Bi Y., Shen H., Zhang L. (2020). Arbuscular Mycorrhizal Fungi Enhance Sea Buckthorn Growth in Coal Mining Subsidence Areas in Northwest China. J. Microbiol. Biotechnol..

[201] Gryndler M., Sudová R., Püschel D., Rydlová J., Janousková M., Vosátka M. (2008). Cultivation of high-biomass crops on coal mine spoil banks: Can microbial inoculation compensate for high doses of organic matter?. Bioresour. Technol..

[202] Kumar A. (2021). Current and Future Perspective of Microalgae for Simultaneous Wastewater Treatment and Feedstock for Biofuels Production. Chem. Afr..

[203] Kumar N. (2014). Extraction of Lipid from Algae Grown in Different open Cast Mining Areas of Jharia Coalfield Under District Dhanbad, Jharkhand: An Experimental Study. Int. J. Curr. Res. Rev..

[204] Hopes A., Mock T. (2015). Evolution of Microalgae and Their Adaptations in Different Marine Ecosystems. eLS.

[205] Shurin J.B., Abbott R.L., Deal M.S., Kwan G.T., Litchman E., McBride R.C., Mandal S., Smith V.H. (2013). Industrial-strength ecology: Trade-offs and opportunities in algal biofuel production. Ecol. Lett..

[206] Roberts D.A., Paul N.A., Bird M.I., de Nys R. (2015). Bioremediation for coal-fired power stations using macroalgae. J. Environ. Manag..

[207] Verb R.G., Vis M.L. (2005). Periphyton Assemblages as Bioindicators of Mine-Drainage in Unglaciated Western Allegheny Plateau Lotic Systems. Water Air Soil Pollut..

[208] O’Neill E.A., Rowan N.J. (2022). Microalgae as a natural ecological bioindicator for the simple real-time monitoring of aquaculture wastewater quality including provision for assessing impact of extremes in climate variance—A comparative case study from the Republic of Ireland. Sci. Total Environ..

[209] Torres M.A., Barros M.P., Campos S.C.G., Pinto E., Rajamani S., Sayre R.T., Colepicolo P. (2008). Biochemical biomarkers in algae and marine pollution: A review. Ecotoxicol. Environ. Saf..

[210] Abdel-Raouf N., Al-Homaidan A.A., Ibraheem I.B.M. (2012). Microalgae and wastewater treatment. Saudi J. Biol. Sci..

[211] Bwapwa J.K., Jaiyeola A.T., Chetty R. (2017). Bioremediation of acid mine drainage using algae strains: A review. S. Afr. J. Chem. Eng..

[212] Santos K.B., Almeida V.O., Weiler J., Schneider I.A. (2020). Removal of Pollutants from an AMD from a Coal Mine by Neutralization/Precipitation Followed by “In Vivo” Biosorption Step with the *Microalgae scenedesmus* sp.. Minerals.

[213] Van Hille R.P., Boshoff G.A., Rose P.D., Duncan J.R. (1999). A continuous process for the biological treatment of heavy metal contaminated acid mine water. Resour. Conserv. Recycl..

[214] Samal D.P.K., Sukla L.B., Pattanaik A., Pradhan D. (2020). Role of microalgae in treatment of acid mine drainage and recovery of valuable metals. Mater. Today Proc..

[215] Dean A.P., Hartley A., McIntosh O.A., Smith A., Feord H.K., Holmberg N.H., King T., Yardley E., White K.N., Pittman J.K. (2019). Metabolic adaptation of a Chlamydomonas acidophila strain isolated from acid mine drainage ponds with low eukaryotic diversity. Sci. Total Environ..

[216] Warjri S.M., Syiem M.B. (2018). Analysis of Biosorption Parameters, Equilibrium Isotherms and Thermodynamic Studies of Chromium (VI) Uptake by a *Nostoc* sp. Isolated from a Coal Mining Site in Meghalaya, India. Mine Water Environ..

[217] Goswami S., Diengdoh O.L., Syiem M.B., Pakshirajan K., Kiran M.G. (2014). Zn (II) and Cu (II) removal by Nostoc muscorum: A cyanobacterium isolated from a coal mining pit in Chiehruphi, Meghalaya, India. Can. J. Microbiol..

[218] Škaloud P., Lukešová A., Malavasi V., Ryšánek D., Hrčková K., Rindi F. (2014). Molecular evidence for the polyphyletic origin of low pH adaptation in the genus Klebsormidium (Klebsormidiophyceae, Streptophyta). Plant Ecol. Evol..

[219] Verb R.G., Vis M.L. (2001). Macroalgal communities from an acid mine drainage impacted watershed. Aquat. Bot..

[220] Das B.K., Roy A., Koschorreck M., Mandal S.M., Wendt-Potthoff K., Bhattacharya J. (2009). Occurrence and role of algae and fungi in acid mine drainage environment with special reference to metals and sulfate immobilization. Water Res..

[221] Freitas A.P.P., Schneider I.A.H., Schwartzbold A. (2011). Biosorption of heavy metals by algal communities in water streams affected by the acid mine drainage in the coal-mining region of Santa Catarina state, Brazil. Miner. Eng..

[222] Phillips P., Bender J., Simms R., Rodriguez-Eaton S., Britt C. (1995). Manganese removal from acid coal-mine drainage by a pond containing green algae and microbial mat. Water Sci. Technol..

[223] Sheoran A.S., Bhandari S. (2005). Treatment of Mine Water by a Microbial Mat: Bench-scale Experiments. Mine Water Environ..

[224] Das M., Ramanujam P. (2011). Metal content in water and in green filamentous algae Microspora quadrata Hazen from coal mine impacted streams of Jaintia Hills district, Meghalaya, India. Int. J. Bot..

[225] Boshoff G., Duncan J., Rose P.D. (2004). The use of micro-algal biomass as a carbon source for biological sulphate reducing systems. Water Res..

[226] Molwantwa J.B., Molipane N.P., Rose P.D. Biological sulfate reduction utilizing algal extracellular products as a carbon source. Proceedings of the WISA 2000 Biennial Conference.

[227] Betts R.A., Jones C.D., Knight J.R., Keeling R.F., Kennedy J.J. (2016). El Niño and a record CO_2_ rise. Nat. Clim. Change.

[228] Giostri A., Binotti M., Macchi E. (2016). Microalgae cofiring in coal power plants: Innovative system layout and energy analysis. Renew. Energy.

[229] Chisti Y. (2007). Biodiesel from microalgae. Biotechnol. Adv..

[230] Hsueh H.T., Chu H., Yu S.T. (2007). A batch study on the bio-fixation of carbon dioxide in the absorbed solution from a chemical wet scrubber by hot spring and marine algae. Chemosphere.

[231] Kumar A., Ergas S., Yuan X., Sahu A., Zhang Q., Dewulf J., Malcata F.X., van Langenhove H. (2010). Enhanced CO_2_ fixation and biofuel production via microalgae: Recent developments and future directions. Trends Biotechnol..

[232] Yahya L., Harun R., Abdullah L.C. (2020). Screening of native microalgae species for carbon fixation at the vicinity of Malaysian coal-fired power plant. Sci. Rep..

[233] De Morais M.G., Costa J.A.V. (2007). Isolation and selection of microalgae from coal fired thermoelectric power plant for biofixation of carbon dioxide. Energy Convers. Manag..

[234] Radmann E.M., Camerini F.V., Santos T.D., Costa J.A.V. (2011). Isolation and application of SOX and NOX resistant microalgae in biofixation of CO2 from thermoelectricity plants. Energy Convers. Manag..

[235] Aslam A., Thomas-Hall S.R., Mughal T.A., Schenk P.M. (2017). Selection and adaptation of microalgae to growth in 100% unfiltered coal-fired flue gas. Bioresour. Technol..

[236] Vaz B.d.S., Costa J.A.V., Morais M.G. (2016). Use of solid waste from thermoelectric plants for the cultivation of microalgae. Braz. Arch. Biol. Technol..

[237] Chen H.-W., Yang T.-S., Chen M.-J., Chang Y.-C., Lin C.-Y., Wang E.I.C., Ho C.-L., Huang K.-M., Yu C.-C., Yang F.-L. (2012). Application of power plant flue gas in a photobioreactor to grow Spirulina algae, and a bioactivity analysis of the algal water-soluble polysaccharides. Bioresour. Technol..

[238] Ikenaga N.-o., Ueda C., Matsui T., Ohtsuki M., Suzuki T. (2001). Co-liquefaction of Micro Algae with Coal Using Coal Liquefaction Catalysts. Energy Fuels.

[239] Corredor L., Barnhart E.P., Parker A.E., Gerlach R., Fields M.W. (2021). Effect of temperature, nitrate concentration, pH and bicarbonate addition on biomass and lipid accumulation in the sporulating green alga PW95. Algal Res..

[240] Kumar N., Banerjee C., Jagadevan S. (2021). Identification, characterization, and lipid profiling of microalgae *Scenedesmus* sp. NC1, isolated from coal mine effluent with potential for biofuel production. Biotechnol. Rep..

[241] Souza L.D., Simioni C., Bouzon Z.L., Schneider R.C., Gressler P., Miotto M.C., Rossi M.J., Rörig L.R. (2017). Morphological and ultrastructural characterization of the acidophilic and lipid-producer strain Chlamydomonas acidophila LAFIC-004 (Chlorophyta) under different culture conditions. Protoplasma.

[242] Xu Y., Yang K., Zhou J., Zhao G. (2020). Coal-Biomass Co-Firing Power Generation Technology: Current Status, Challenges and Policy Implications. Sustainability.

[243] Chen C., Ma X., He Y. (2012). Co-pyrolysis characteristics of microalgae Chlorella vulgaris and coal through TGA. Bioresour. Technol..

[244] Miranda M.T., Sepúlveda F.J., Arranz J.I., Montero I., Rojas C.V. (2018). Physical-energy characterization of microalgae Scenedesmus and experimental pellets. Fuel.

[245] Yoo C., Jun S.-Y., Lee J.-Y., Ahn C.-Y., Oh H.-M. (2010). Selection of microalgae for lipid production under high levels carbon dioxide. Bioresour. Technol..

[246] Baloyi H., Dugmore G. (2019). Influences of microalgae biomass on the thermal behaviour of waste coal fines. J. Energy S. Afr..

[247] Ejesieme V.O., Vorster N., Riaza J., Dugmore G., Zeelie B. (2020). Reclamation of ultra-fine coal with scenedesmus microalgae and comprehensive combustion property of the Coalgae^®^ composite. J. Energy S. Afr..

[248] Magida N.E., Bolo L.L., Hlangothi S.P., Dugmore G., Ogunlaja A.S. (2021). Co-combustion Characteristics of coal-Scenedesmus Microalgae Blends and Their Resulting Ash. Combust. Sci. Technol..

[249] Nyoni B., Duma S., Bolo L., Shabangu S., Hlangothi S.P. (2020). Co-pyrolysis of South African bituminous coal and Scenedesmus microalgae: Kinetics and synergistic effects study. Int. J. Coal Sci. Technol..

[250] Wu Z., Yang W., Tian X., Yang B. (2017). Synergistic effects from co-pyrolysis of low-rank coal and model components of microalgae biomass. Energy Convers. Manag..

[251] Subagyono R.R.D.J.N., Masdalifa W., Aminah S., Nugroho R.A., Mollah M., Londong Allo V., Gunawan R. (2021). Kinetic Study of Copyrolysis of the Green Microalgae Botryococcus braunii and Victorian Brown Coal by Thermogravimetric Analysis. ACS Omega.

[252] Sanchez-Silva L., López-González D., Garcia-Minguillan A.M., Valverde J.L. (2013). Pyrolysis, combustion and gasification characteristics of Nannochloropsis gaditana microalgae. Bioresour. Technol..

[253] Tahmasebi A., Kassim M.A., Yu J., Bhattacharya S. (2013). Thermogravimetric study of the combustion of Tetraselmis suecica microalgae and its blend with a Victorian brown coal in O_2_/N_2_ and O_2_/CO_2_ atmospheres. Bioresour. Technol..

[254] Agrawal A., Chakraborty S. (2013). A kinetic study of pyrolysis and combustion of microalgae Chlorella vulgaris using thermo-gravimetric analysis. Bioresour. Technol..

[255] Hossain N., Zaini J., Mahlia T.M.I., Azad A.K. (2019). Elemental, morphological and thermal analysis of mixed microalgae species from drain water. Renew. Energy.

